# EZH2 inhibitors reverse resistance to gefitinib in primary EGFR wild-type lung cancer cells

**DOI:** 10.1186/s12885-020-07667-7

**Published:** 2020-12-04

**Authors:** Hao Gong, Yongwen Li, Yin Yuan, Weiting Li, Hongbing Zhang, Zihe Zhang, Ruifeng Shi, Minghui Liu, Chao Liu, Chen Chen, Hongyu Liu, Jun Chen

**Affiliations:** 1 Department of Lung Cancer Surgery, Tianjin Medical University General Hospital, Tianjin, P.R. China; 2 Tianjin Key Laboratory of Lung Cancer Metastasis and Tumor Microenvironment, Tianjin Lung Cancer Institute, Tianjin Medical University General Hospital, Tianjin, 300052 P.R. China

**Keywords:** Non–small-cell lung cancer, Enhancer of zeste homolog 2 (EZH2), EZH2 inhibitor, EGFR-TKIs

## Abstract

**Background:**

Lung cancer is the leading cause of cancer-related deaths worldwide. Non-small cell lung cancer (NSCLC) is the most common type of lung cancer. In traditional anti-cancer therapy, epidermal growth factor receptor (EGFR)-tyrosine kinase inhibitors (TKI) have been proven to be beneficial for patients with EGFR mutations. However, patients with EGFR wild-type NSCLC were usually not respond to EGFR-TKIs. Enhancer of zeste homolog 2 (EZH2) is a key molecular in the PRC2 complex and plays an important role in epigenetic regulation and is overexpressed in variant tumors. EZH2 inhibitors have been reported to sensitize variant tumor cells to anticancer drugs. This study aimed to investigate whether the EZH2 inhibitors, GSK343 and DZNep when combined with gefitinib can reverse EGFR-TKIs resistance in EGFR wild-type NSCLC cells.

**Methods:**

The RNA-sequencing data of patients with NSCLC [502 patients with lung squamous cell carcinoma, including 49 paracancerous lung tissues and 513 patients with lung adenocarcinoma (LUAD), including 59 paracancerous lung tissues] from the Cancer Genome Atlas (TCGA), were analyzed for EZH2 expression. EZH2 expression was verified in 40 NSCLC tissue cancer samples and their corresponding paracancerous tissues from our institute (TJMUGH) via RT-PCR. A549 and H1299 cells treated with siRNA or EZH2 inhibitors were subjected to cell viability and apoptosis analyses as well to EGFR pathway proteins expression analyses via western blotting.

**Results:**

EZH2 was upregulated in human NSCLC tissues and correlated with poor prognosis in patients with LUAD based on data from both TCGA and TJMUGH. Both GSK343 and DZNep sensitized EGFR wild-type LUAD cells (A549 and H1299) to gefitinib and suppressed cell viability and proliferation in vitro by downregulating the phosphorylation of EGFR and AKT and by inducing cell apoptosis. Co-administration of EZH2 inhibitors (GSK343 or DZNep) with gefitinib exerted a stronger inhibitory effect on tumor activity, cell proliferation and cell migration than single drug administration in vitro and in vivo.

**Conclusions:**

These data suggest that the combination of EZH2 inhibitors with EGFR-TKIs may be an effective method for treating NSCLC-patients with EGFR-wild type, who do not want to undergo traditional treatment with chemotherapy.

## Background

Lung cancer is the leading cause of cancer-related death worldwide and is characterized by early metastasis, high mortality, and poor survival [1].Non–small-cell lung cancer (NSCLC),including lung adenocarcinoma (LUAD), squamous cell carcinoma (LUSC), and large-cell carcinoma accounts for approximately 85% of all lung cancer cases [2]. Despite advances in the clinical diagnosis of lung cancer and the associated therapeutic strategies, patients with late-stage disease have a 5-year overall survival (OS) rate of only 11–16% [2, 3]. The lack of biomarkers to facilitate the diagnosis of early-stage disease and cancer metastasis remains one of critical challenges in NSCLC therapy [4]. Therefore, a profound understanding of the molecular mechanisms contributing to the development and progression of NSCLC is essential for developing specific diagnostic methods,as well as for designing individualized and effective physiological strategies.

Targeted drugs such as epidermal growth factor receptor (EGFR)-tyrosine kinase inhibitors (EGFR-TKIs) represent one of the vital advances in lung cancer treatment. EGFR is an essential receptor tyrosine kinase that can regulate cell proliferation and differentiation, and its abnormal activation contributes to a variety of human cancers [5]. EGFR signaling activated constitutively by a gene mutation, gene amplification, or both, and this event has been shown to be closely related to the occurrence, progression, and poor prognosis of NSCLC [6, 7]. The EGFR-driven mutation rate is 15% in the Caucasian population and 40–62% in the Asian population [8]. The discovery of EGFR-activating mutations in NSCLC and the successful use of EGFR-TKIs have shifted the focus of cancer treatment from empirical cytotoxic chemotherapy to molecularly targeted therapies. The current common practices involve the administration of EGFR-TKIs as first-line treatment to patients with EGFR-sensitive mutations because these drugs have been shown to considerably prolong progression-free survival while causing fewer adverse effects than chemotherapy [9].EGFR-TKIs have also been approved as the second- or third-line treatment for EGFR wild-type (EGFR-WT) NSCLC. However, the use of these targeted drugs beyond first-line treatment remains controversial, particularly for the treatment of EGFR-WT NSCLC.

Recently,histone post-translational modifications such as acetylation, methylation, and phosphorylation have been found to play important roles in tumorigenesis [8]. Histones are considered important centers of epigenetic regulation. Polycomb group proteins such as PCR2 act as transcriptional repressors by silencing specific sets of genes via chromatin modification,thereby playing key roles in various epigenetic phenomena. Enhancer of zeste homolog 2 (EZH2), a key component of polycomb suppression complex 2 (PRC2), is responsible for the monomethylation, dimethylation, and trimethylation of histone H3K27 [10]. EZH2 overexpression has been described in various human cancers including NSCLC [11–13]. EZH2 can also promote the development and progression of cancer via chromatin modification including the epigenetic activation of the oncogenic signaling cascade and silencing of tumor suppressor genes; it has been implicated in cell proliferation, differentiation, invasion, and metastasis [11, 14, 15]. EZH2 overexpression is associated with poor prognosis in lung cancer; therefore, it is considered an attractive therapeutic target [16, 17]. In addition, various EZH2 inhibitors have been developed and the safety and anticancer efficacy of these drugs are being investigated in ongoing research and clinical studies. The commonly used EZH2 inhibitors can be classified into two types. The first type comprises S-adenosyl-L-homocysteine (SAH) hydrolase inhibitors, which block the hydrolysis of SAH into homocysteine and adenosine and indirectly inhibit the methionine cycle and S-adenosyl-L-methionine (SAM) regeneration, resulting in the consumption of EZH2 via the bypass route. 3-DeazaneplanocinA (DZNep) is the most common representative of this drug type [18]. The second type of EZH2 inhibitor comprises competitive SAM inhibitors. These drugs compete with SAM for binding sites on H3K27, thereby decreasing the number of bound SAMs. In turn, the activities of SAM-dependent methyltransferases are inhibited and the trimethylation of H3K27 is blocked. GSK343 and GSK126 are the most well-known representatives of this drug type.

EZH2 is overexpressed in various tumors including lung cancer. EZH2 inhibitors have been reported to sensitize many types of tumor cells to antitumor drugs. In the present study, we investigated the role of EZH2 inhibitors in reversing acquired resistance to gefitinib in EGFR-WT NSCLC cells. We demonstrated that EZH2 was upregulated in human NSCLC tissues and that this upregulation was correlated with poor prognosis in patients with LUAD. GSK343 and DZNep both sensitized EGFR-WT LUAD cells (A549 and H1299) to gefitinib and suppressed cell viability and proliferation in vitro by downregulating the phosphorylation of EGFR and AKT and inducing cell apoptosis. The co-administration of these EZH2 inhibitors with gefitinib exerted stronger inhibitory effects on tumor activity than the administration of either drug alone.

## Methods

### Patients and tissue specimens

A total of 40 paired NSCLC and their paracancerous lung tissues were obtained from patients who were diagnosed with NSCLC based on histopathological evaluations and had undergone surgery at Tianjin Medical University General Hospital (TJMUGH) between January 2010 and December 2011. Among these, 16 patients were diagnosed with primary LUAD and 24 with primary LUSC. In each patient, lung cancer staging was performed according to the AJCC Cancer Staging Manual, 8th edition, and findings of physical examination; surgical resection; and computed tomography of the chest, abdomen, pelvis, and brain. All collected tissue samples were immediately snap-frozen in liquid nitrogen and stored at − 80 °C prior to RNA extraction. Basic demographic and clinical information such as sex, age, smoking history, TNM stage, lymph node metastasis, and prognostic data were also collected from medical records. The ethics committee of TJMUGH approved this study.

### Downloading of the Cancer genome atlas (TCGA) data and processing of RNA-seq data

A cohort of 1015 human lung cancer specimens (including 513 LUAD, including 59 paracancerous lung tissues, and 502 LUSC, including 49 paracancerous lung tissues) from the Cancer Genome Atlas (TCGA) database (https://cancergenome.nih.gov/) was used to evaluate EZH2 expression levels in lung cancer. Transcripts per million (TPM) was calculated and normalized using the Tag count comparison package (version 1.6.5; http://www.bioconductor.org/ packages/release/bioc/html/ TCC.html) [19]. The Kaplan-Meier method and log-rank test were used to evaluate the correlations between EZH2 expression and OS patients with LUAD(*n* = 352) and LUSC(*n* = 409).

### Cells culture

The human LUAD cell lines A549 and HCC827 cells were purchased from the American Type Culture Collection (Manassas, VA, USA). Other human LUAD cell lines (H460, H1299, H1975, HCC827, H1650, and H2030) and the normal human bronchial epithelial cell line BEAS-2B were obtained from the Institute of Biochemistry and Cell Biology of the Chinese Academy of Sciences (Shanghai, China). The H460 and A549 cell lines harbor a *KRAS* mutation; HCC827 and H1650 harbor an *EGFR* exon 19 deletion; H1299 harbors a p53 deletion or rearrangement mutation; H1975 harbors *EGFR* exon 21 mutation (L858R) and exon 20 (T790M) mutations; H1792 harbors a *TP53* splice mutation and *EGFR* exon 21 (L858R) and exon 20 (T790M) mutations; and H2030 harbors *EGFR* exon 21 (L858R) and *KRAS* mutations. All cell lines were maintained in RPMI 1640 medium supplemented with 10% fetal bovine serum (FBS), 100 IU/ml penicillin, and 100 μg/ml streptomycin (Invitrogen, Carlsbad, CA, USA). All cells were cultured at 37 °C in a humidified atmosphere containing 5% CO_2_.

### Transfection and drug treatment

Cells were seeded into a well of 6-well plate and were cultured to approximately 60% confluency. Subsequently,the cells were transfected with EZH2 siRNA oligonucleotides and non-targeting siRNA (Guangzhou, China) using Lipofectamine 2000 (Invitrogen) according to the manufacturer’s instructions. Cells in the exponential growth phase were then incubated with phosphate-buffered saline (PBS) (NC group) or various concentrations of gefitinib (ZD1839), GSK343, and DZNep HCl (Selleck Chemicals LCC, USA) for 48 h.The cells were then harvested for downstream experiments.

### Cell counting Kit-8 (CCK-8) assay

The CCK-8 assay (Beyotime,Shanghai,China) was performed according to the manufacturer’s instructions to measure of cell proliferation and to determine the half-maximal inhibitory concentration (IC_50_) of each drug in each cell line. Briefly, 5000 cells were seeded in a 96-well plate and incubated overnight. The cells were then treated with different concentrations of gefitinib, GSK343, DZNep, GSK + gefitinib (G + g), and DZNep+gefitinib (D + g) for 48 h. Next, 10 μl CCK-8 (5 mg/ml) was added to each well and the cultures were incubated at 37 °C for 1 h. The absorbance was measured at 450 nm using a microplate reader (SpectraMax M5, Molecular Devices, Sunnyvale, CA, USA). The experiments were repeated at least three times.

### 5-Ethynyl-2′-deoxyuridine (EdU) staining

The Cell-Light™ EdU staining (RiboBio,Guangzhou,China) was used to measure cell proliferation according the manufacturer’s instructions. Briefly, cells were incubated with 50 μM EdU for 2 h, followed by two washes with PBS, then the cells were fixed with 4% paraformaldehyde. After penetration with 0.5% Triton X-100 and washing with PBS, the cells were dyed with Apollo (Red) and Hoechst 33342 (Blue) in the dark for 30 min. Stained cells were visualized under a fluorescence microscope.

### Colony formation assay

The colony formation assay was performed to study the inhibitory effect of each drug in NSCLC cells. Briefly, 500 cells were seeded into each well of a 6-well plate and incubated at 37 °C for 24 h. Then the cells were treated with compounds (gefitinib, GSK343, DZNep, G + g, and D + g) and the medium was replaced with fresh medium every 3 days. After approximately 14 days, the colonies were fixed in methanol and stained with 0.5% crystal violet at room temperature for 30 min. The number of colonies (defined as > 50 cells) was scored and photographed.

### Flow cytometry analysis of apoptosis and cell cycle

Cells (2 × 10^5^ cells/well) were seeded into 6-well plates and cultured for 24 h. The compounds (NC, GSK343, DZNep, gefitinib, G + g, and D + g) were added at various indicated concentrations for 48 h. For the cell apoptosis assay, cells were stained using the Annexin V-FITC Apoptosis Analysis Kit (BD Biosciences, San Jose, CA, USA) and were analyzed using the FACSAria™ flow cytometer (BD Biosciences). For the cell cycle assay, cells were trypsinized and fixed with 70% ice-cold ethanol overnight. Subsequently, cells were treated with DNase-free ribonuclease (TaKaRa, Beijing, China), stained with propidium iodide (PI; BD Biosciences), and analyzed using the FACSAria™ flow cytometer (BD Biosciences) equipped with ModFit LT (Topsham, ME, USA).

### Wound healing assay

H1299 cells were seeded into 6-well plates. After cells had grown to 90–100% confluency, the cross lines were introduced using a 200-μL sterile pipette tip. After scratching,the suspended cells were removed gently with PBS and were incubated in RPMI 1640 medium with 2% FBS for 48 h. Finally, the images were captured under a microscope. Wound width was calculated as the gap distance at 48 h/gap distance at 0 h.. The results of three independent experiments were averaged.

### Transwell invasion assay

The invasion assay was performed using Transwell chambers (Corning,NY,USA). Briefly, the upper surfaces of the polycarbonic membranes were coated with 100 μl of 300 μg/mL Matrigel and then placed in the chambers at 37 °C for 30 min. Next, 1 × 10^5^ cells were seeded in each upper chamber with the indicated doses of compounds in starvation medium, and 600 μL medium containing 20% FBS was added to each lower chamber. After incubation for 24 h, the invaded cells were fixed with methanol and stained with 0.1% crystal violet at room temperature for 10 min. The Nikon TE2000 microscope (Tokyo, Japan) at 100× magnification was used to collect five randomly selected visual field images. The experiments were performed in triplicate.

### In vivo study

Female BALB/c athymic nude mice (4–5 weeks old) were purchased from the Experimental Laboratory Animal Center of Beijing University (Beijing, China) and were housed in the animal facilities of Tianjin Medical University. All mice were maintained under specific pathogen-free conditions and were examined prior to starting the study to ensure that they were healthy and could be adapted for tumor implantation. A549 cells (2 × 10^6^) were injected subcutaneously injected into the left flanks of nude mice. When the tumors reached a volume of approximately 200 mm^3^, the mice were randomly distributed into six groups (10 mice/group): vehicle group (administered 0.5% methylcellulose 400 and sodium lactate buffer), GSK343 group (administered 4 mg/kg GSK343 in 100 μL of corn oil), DZNep group (2 mg DZNep/kg in 100 μL of corn oil), gefitinib group (administered 100 mg/kg gefitinib/day), G + g group (administered 4 mg/kg GSK343 in 100 μL corn oil plus 100 mg/kg gefitinib/day), and D + g group (administered 2 mg/kg DZNep in 100 μL corn oil). Tumor size was measured every alternate day. Tumor volume (V) was calculated using the following formula: Volume (mm^3^) = (Length × Width^2^) × 0.5 ^[24]^ After 4 weeks, the mice were injected with Barbiturate at 100 mg/kg for euthanasia and the tumors were harvested and weighed. The tumors isolated from mice were then preserved in 4% paraformaldehyde at 4 °C until future analysis. In vivo studies were conducted as per the institutional ethics guidelines for animal experiments, which were accepted by the Animal Management Committee of the Tianjin Medical University.

### Immunohistochemistry (IHC)

Xenograft tumor tissues were fixed overnight in 4% formalin, dehydrated with ethanol, and embedded in paraffin. Next, 4 μm-thick tissue slices were first dewaxed with xylose and rehydrated using an alcohol solution gradient. After a 10-min blocking step with normal goat serum, the tissue slices were incubated with a primary antibody specific for EZH2 (Cell Signaling Technology, MA, USA. 1:400 dilution), Ki67 (Bioss, Beijing, China.1:200 dilution) and Caspase 3 (Bioss, Beijing, China.1:200 dilution) for 1 h at room temperature, washed with PBS, and incubated with a horseradish peroxidase (HRP)-conjugated secondary antibody (ZSJQ Corp, Beijing, China) for 60 min. Finally, the sections were incubated with 3,3′-diaminobenzidine for 3 min at room temperature and counter stained with hematoxylin.

### RNA extraction and quantitative PCR (qPCR) assays

Total RNA was extracted from patient tissue samples or cultured cells using the TRIzol reagent (Invitrogen) according to the manufacturer’s instructions. RNA was quantified using a spectrophotometer (Beckman, USA), and RNA quality was assessed using denaturing 1.2% agarose gel electrophoresis. Subsequently, 2 μg of extracted RNA was reverse transcribed in a final reaction volume of 20 μl using random primers under standard conditions specified in the PrimeScript RT reagent Kit (TaKaRa, Dalian,China) and reverse transcriptase (Promega, Beijing, China). Real-time PCR was performed using the Power SYBR Green PCR Master Mix (Applied Biosystems, Foster City, CA, USA) according to the manufacturer’s instructions on a ABI 7500 real-time PCR system (Applied Biosystems). The expression levels of EZH2 and EGFR were normalized to those of glyceraldehyde-3-phosphate dehydrogenase (GAPDH) using 2^−ΔΔCT^ method. All gene primers were obtained from SBS Genetech (Beijing, China).

### Western blotting

Cells were collected and lysed in RIPA buffer [(1× PBS, 1% NP40, 0.5% sodium deoxycholate, 0.1% sodium dodecyl sulfate (SDS)) supplemented with 2 mM PMSF (Thermo Fisher Scientific, Inc., Waltham, MA, USA). The BCA protein assay kit (Pierce Biotechnology, Rockford, IL, USA) was used to measure protein concentrations. Next, equal amounts of proteins (30 μg) were separated using 10% SDS-polyacrylamide gels, followed by transferring the gels onto polyvinylidene fluoride membranes (Millipore, Lake Placid, NY, USA). The membranes were blocked with 5% non-fat milk and incubated overnight at 4 °C with dilutions of the following primary antibodies: rabbit anti-EZH2 (dilution, 1:1000; Cell Signaling Technology), mouse anti-β-actin (1:3000; Sigma-Aldrich, St. Louis, MO, USA), rabbit anti-EGFR (1:1000; Cell Signaling Technology), rabbit anti-phospho (p)-EGFR (1:1000; Cell Signaling Technology), rabbit anti-AKT (1:1000; Cell Signaling Technology), rabbit anti-p-AKT (1:1000; Cell Signaling Technology), rabbit anti-P38-MAPK (1:1000; Cell Signaling Technology), rabbit anti-p-P38-MAPK (1:1000; Cell Signaling Technology), rabbit anti-BCL-2 (1:1000; Cell Signaling Technology), rabbit anti-Bax (1:1000; Cell Signaling Technology), rabbit anti- caspase-3 (1:1000; Cell Signaling Technology), rabbit anti-P62 (1:1000; Cell Signaling Technology),rabbit anti-LC3B (1:800; Cell Signaling Technology), rabbit anti-Vimitin (1:1000; Cell Signaling Technology), mouse anti-N-cadherin (1:1000; Cell Signaling Technology), rabbit anti-Cleaved PARP (1:400; Beijing Bioss biotechnology) and rabbit anti-MMP2 (1:400; Beijing Bioss biotechnology). Subsequently, the membranes were exposed to an HRP-conjugated secondary antibody (1:1000 dilution; Thermo Fisher Scientific, Inc.) at room temperature for 1 h. Finally, the Pierce ECL Substrate (Thermo Fisher Scientific, Inc.) was used to visualize the bands.

### Statistical analysis

Statistical analysis was performed using the SPSS software package, version 21.0 (IBM Corp., Armonk, NY, USA). Data are presented as mean ± standard deviation of independent experiments. Kaplan–Meier survival analysis was performed to estimate OS. Univariate cox regression analysis and subsequent multivariate analysis using the likelihood ratio were performed to identify significant variables. The statistical significance of differences between two groups was analyzed using the Student’s *t*-test. All *P*-values obtained in this study were two tailed, and the statistical significance level was set at *P*-value of < 0.05.

## Results

### EZH2 is strongly expressed in primary lung cancer tissues

**Fig. 1:**
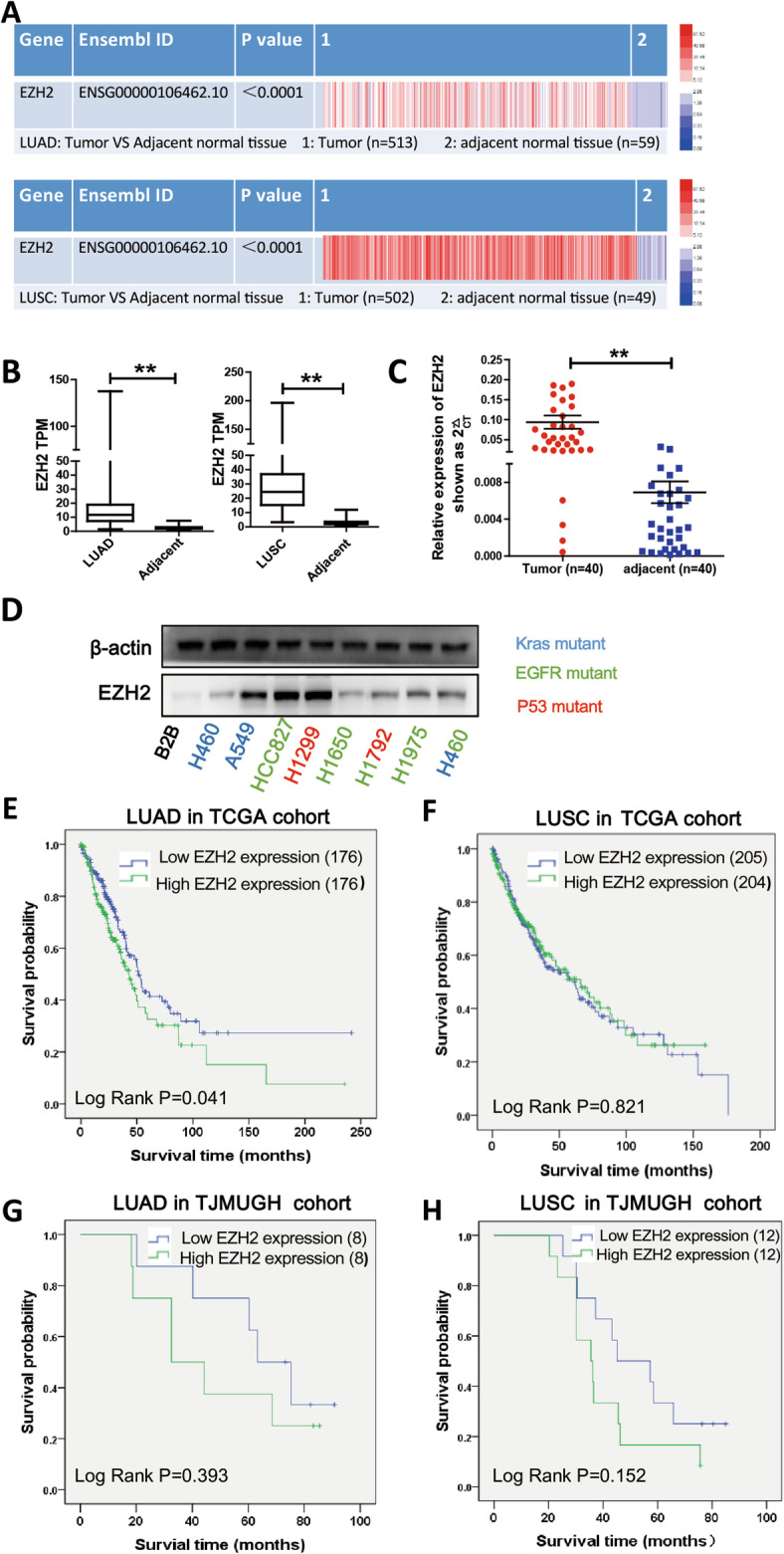
EZH2 is high expressed in non–small-cell lung cancer (NSCLC) and is associated with poor prognosis in patients with lung adenocarcinoma. **a** Relative expression of EZH2 in NSCLC tissues compared with in paracancerous lung tissues in The Cancer Genome Atlas dataset. **b** EZH2 TPM in NSCLC and paracancerous lung tissues in TCGA dataset. **c** EZH2 expression levels in NSCLC tissues (*n* = 40) compared with those in paracancerous lung tissues (*n* = 40) by qPCR analysis; data were normalized against GAPDH expression level. **d** EZH2 protein levels in different NSCLC lines and the normal alveolar epithelial cell line BEAS-2B. **e**-**f** Overall survival curves of patients with LUAD and LUSC from the TCGA cohort. **g**–**h** Overall survival curves of patients with LUAD and LUSC from the TJMUGH cohort

EZH2 overexpression has been reported in multiple tumor types. To determine of EZH2 expression level in NSCLC tumor tissues compared with that in paracancerous lung tissues, LUAD tissues from 513 patients (including 59 paired tumor and paracancerous lung tissues) and LUSC tissues from 502 patients (including 49 paired tumor and paracancerous lung tissues) were collected from the TCGA database. The original analysis of RNA expression indicated considerably higher EZH2 expression level in lung cancer tissues than in the paracancerous lung tissues (*P* < 0.01, Fig. 1a). As shown in Fig. 1b, the TPM of EZH2 (15.55 ± 13.28) was much higher in LUAD tissues than in the paracancerous lung tissues (2.53 ± 1.11; *P* < 0.001); similarly, the TPM of EZH2 (28.57 ± 19.49) was much higher in LUSC tissues than in the paracancerous lung tissues (2.94 ± 1.75; *P* < 0.01). To verify the results of TCGA data analysis, we extracted RNA from NSCLC and their paracancerous lung tissues collected from the TJMUGH cohort and verified EZH2 expression level via qPCR. Notably, the EZH2 expression level in NSCLC tissues (*n* = 40; 2^−ΔΔCT^ = 0.09 ± 0.1) was much higher than that in paracancerous lung tissues (2^−ΔΔCT^ = 0.01 ± 0.01; *P* < 0.01, Fig. 1c). Western blotting revealed higher EZH2 expression level in NSCLC cell lines, particularly in A549, HCC827, and H1299, than in the normal bronchus epithelial cell line BEAS-2B (Fig. 1d).

### Correlation between EZH2 expression level and clinicopathological characteristics in NSCLC

To investigate the correlation between the EZH2 expression level and clinicopathological characteristics of NSCLC, we analyzed the data of 352 patients with LUAD and 409 with LUSC from the TCGA cohort and 40 patients with NSCLC from the TJMUGH cohort. A correlation was found between EZH2 expression level and T stage in patients with LUAD. Higher EZH2 expression levels were observed in smaller tumors (T1–T2) than in larger tumors (*P* = 0.033, Table 1). EZH2 expression was correlated with a history of smoking (*P* = 0.012) in patients with LUAD. However, no other correlations were observed between EZH2 expression level and other clinicopathological characteristics of patients with LUAD such as age, sex, lymphatic metastasis status, distant metastasis, TNM stage, and tumor recurrence status. Moreover, we observed no significant associations between EZH2 expression and the clinicopathological characteristics of patients with LUSC. We obtained similar results in an analysis of 40 patients with NSCLC from the TJMUGH cohort, irrespective of the NSCLC subtype (Table 1).

**Table 1:** Clinicopathological characteristics of NSCLC patients based on the EZH2 expression in TCGA and TJMUGH cohort

	LUAD in TCGA cohort (*n* = 352)			LUAD in TJMUGH cohort (*n* = 16)			LUSC in TCGA cohort (*n* = 409)			LUSC in TJMUGH cohort (*n* = 24)		
Characteristics	Low EHZ2 level (*n* = 176)	High EHZ2 level (*n* = 176)	X2 test (*P*-value)	Low EHZ2 level (*n* = 8)	High EHZ2 level (*n* = 8)	X2 test (*P*-value)	Low EZH2 level (*n* = 205)	High EHZ2 level (*n* = 204)	X2 test (*P*-value)	Low EZH2 level (*n* = 12)	High EHZ2 level (*n* = 12)	X2 test (*P*-value)
**Age (%)**												
≤ 65	73 (41.5)	93 (52.8)	0.098	4 (50)	2 (25)	0.608	84 (41.0)	77 (37.7)	0.225	5 (41.7)	10 (83.3)	0.089
> 65	97 (55.1)	79 (44.9)		4 (50)	6 (75)		120 (58.5)	122 (59.8)		7 (58.3)	2 (16.7)	
Unknown	6 (3.4)	4 (2.3)					1 (0.5)	5 (2.5)				
**Gender (%)**												
Male	85 (48.3)	90 (51.1)	0.670	6 (75)	4 (50)	0.608	156 (76.1)	149 (73.0)	0.497	10 (83.3)	11 (73.0)	1.000
Female	91 (51.7)	86 (48.9)		2 (25)	4 (50)		49 (23.9)	55 (27)		2 (16.7)	1 (27)	
**T stage (%)**												
T1-T2	143 (81.3)	125 (71.0)	0.033	6 (75)	6 (75)	1.000	172 (83.9)	162 (79.4)	0.253	11 (91.7)	8 (66.7)	0.317
T3-T4	33 (16.5)	51 (29.0)		2 (25)	2 (25)		33 (16.1)	42 (20.6)		1 (8.33)	4 (33.3)	
**Lymph node metastasis (%)**												
Without	116 (65.9)	106 (60.2)	0.320	4 (50)	4 (50)	1.000	131 (63.9)	129 (63.2)	0.918	7 (58.3)	8 (66.7)	0.414
With	60 (18.8)	70 (39.8)		4 (50)	4 (50)		74 (36.1)	75 (36.8)		5 (41.7)	4 (33.3)	
**Distant metastasis (%)**												
No distant metastasis	168 (95.5)	162 (92)	0.186	8 (100)	8 (100)		200 (97.6)	203 (99.5)	0.215	12 (100)	12 (100)	
Distant organs metastasis	8 (4.5)	14 (8.0)					5 (2.4)	1 (0.5)				
**TNM stage (%)**												
I + II	105 (96.3)	108 (99.1)	0.116	5 (62.5)	5 (62.5)	1.000	165 (80.5)	167 (81.9)	0.409	8 (66.7)	7 (58.3)	0.414
III + IV	4 (3.7)	1 (0.9)		3 (37.5)	3 (37.5)		40 (19.5)	37 (18.1)		4 (33.3)	5 (41.7)	
**Tumor recurrence (%)**												
With tumor	34 (19.3)	37 (21.0)	0.733	2 (75)	1 (12.5)	1.000	45 (22.0)	32 (15.7)	0.240	4 (33.3)	2 (16.7)	0.640
Tumor free	99 (56.3)	102(58.0)		6 (25)	7 (87.5)		99 (8.3)	102 (50)		8 (66.7)	10 (83.3)	
Unknown	43 (24.4)	37 (21.0)					61 (29.8)	70 (34.3)				
**Smoker history (%)**												
Never smokers	26 (14.8)	24 (13.6)	0.012	5 (62.5)	5 (62.5)	0.619	7 (3.4)	8 (3.9)	0.963	5 (41.6)	2 (16.7)	0.371
Smokers	139 (79)	151 (85.8)		3 (37.5)	3 (37.5)		192 (93.7)	190 (93.1)		7 (58.3)	10 (83.3)	
Unknown	11(6.3)	1(0.6)					6 (2.9)	6 (2.9)				

*LUAD* lung adenocarcinoma, *LUSC* Lung squamous cell carcinoma, *TCGA* The Cancer Genome Atlas.

### High **EZH2** expression level is associated with poor prognosis in LUAD

Next, overall survival (OS) was estimated using Kaplan–Meier survival analysis and compared using the log-rank test to analyze the associations between EZH2 expression level and OS outcomes. Analysis of TCGA data revealed that among patients with LUAD, the high EZH2 expression group had a much lower OS than the low EZH2 expression group (log-rank test, *P* = 0.041; Fig. 1e). Univariate analysis identified EZH2 expression, age, T stage, lymph node metastasis, distant metastasis, TNM classification, and tumor recurrence as independent prognostic factors of OS in patients with LUAD (log-rank test, *P* = 0.041, 0.026, < 0.01, < 0.01, 0.038, < 0.01, and < 0.01, respectively; Table 2). No significant correlations were observed between OS and other factors such as sex and smoking status. In patients with LUSC, tumor recurrence status, TNM classification, and smoking status were identified as independent prognostic factors for OS (log-rank test, *P* = 0.020, 0.01 and 0.048, respectively). Multivariate analysis identified EZH2 expression, age, T stage, lymph node metastasis, and tumor recurrence to be significantly correlated with prognosis in patients with LUAD (log-rank test, *P* = 0.048, 0.031, 0.026, < 0.01, and < 0.01, respectively), whereas only tumor recurrence was identified to be significantly correlated with prognosis in patients with LUSC (log-rank test, *P* < 0.01). Taken together, these results indicate that EZH2 expression level is correlated with prognosis. As shown in Table 3, the mean OS and median OS in the low EZH2 expression group was 96.95 and 50.93 months, respectively. The mean and median OS in the high EZH2 expression group was 65.88 and 43.93 months, respectively. However, in patients with LUSC, EZH2 expression was not significantly correlated with prognosis (Fig. 1f, log-rank test, *P* = 0.821). The mean OS and median OS were 75.31 and 61.37 months in the low EZH2 expression group and 74.62 and 65.83 months in the high EZH2 expression group, respectively (Table 3).

**Table 2:** Univariate and multivariate Cox hazard regression in TCGA cohort

Pathological type	Characteristics	Univariate analysis Hazard Ratio (95%CI)	*P* value	Multivariate analysis Hazard Ratio (95%CI)	*P* value
LUAD	EZH2 expression	1.412 (1.013–1.967)	0.041	1.386 (0.976–1.968)	0.048
	Age	1.013 (1.001–1.024)	0.026	1.029 (0.748–1.417)	0.031
	Gender	0.951 (0.684–1.323)	0.767	1.043 (0.744–1.463)	0.807
	T stage	2.619 (1.743–3.936)	0.000	1.753 (1.069–2.872)	0.026
	Lymph node metastasis	2.661 (1.906–3.713)	0.000	2.330 (1.567–3.464)	0.000
	Presence of distant metastasis	1.936 (1.092–3.434)	0.038	1.215 (0.630–2.343)	0.560
	TNM classification	2.775 (1.968–3.913)	0.000	1.365 (0.838–2.225)	0.212
	Tumor recurrence	1.315 (1.167–1.482)	0.000	1.282 (1.131–1.454)	0.000
	Smoker status	1.083 (0.826–1.419)	0.569	0.930 (0.689–1.255)	0.635
LUSC	EZH2 expression	0.966 (0.717–1.302)	0.821	0.956 (0.703–1.299)	0.773
	Age	1.196 (0.898–1.594)	0.219	1.306 (0.966–1.765)	0.083
	Gender	0.718 (0.497–1.037)	0.069	0.765 (0.528–1.109)	0.157
	T stage	1.451 (1.004–2.096)	0.056	1.111 (0.716–1.724)	0.639
	Lymph node metastasis	1.098 (0.808–1.491)	0.552	0.900 (0.623–1.301)	0.576
	Presence of distant metastasis	2.506 (0.925–6.791)	0.114	1.839 (0.632–5.351)	0.264
	TNM classification	1.546 (1.085–2.203)	0.020	1.326 (0.800–2.197)	0.274
	Tumor recurrence	1.369 (1.229–1.524)	0.000	1.343 (1.202–1.500)	0.000
	Smoker status	0.619 (0.366–1.048)	0.048	0.765 (0.528–1.109)	0.127

**Table 3:** EZH2 expression levels associated with NSCLC overall survival in TCGA cohort and TJMUGH cohort。

	Pathological type	Factors	n	Mean survival (Months)	Median survival (Months)	Univariate analysis (*P* value)
TCGA chort	LUAD	Low EZH2 expression	176	96.95	50.93	0.041
		High EZH2 expression	176	65.88	43.93	
	LUSC	Low EZH2 expression	205	75.31	61.37	0.821
		High EZH2 expression	204	74.62	65.83	
TJMUGH chort	LUAD	Low EZH2 expression	8	65.77	63.25	0.393
		High EZH2 expression	8	48.22	32.54	
	LUSC	Low EZH2 expression	12	54.03	45.21	0.152
		High EZH2 expression	12	40.48	35.57	

However, clinical data analysis did not identify a significant difference in OS between the high and low EZH2 expression groups for LUAD or LUSC in TJMUGH cohort (log-rank test, *P* = 0.393 and 0.152, respectively; Fig. 1g and h). However, patients with either type of cancer in the high EZH2 expression group tended to have shorter median and mean OS than those in the low EZH2 expression group (48.22 and 32.54 months vs. 65.77 and 63.25 months in LUAD, respectively; 40.48 and 35.57 months vs 54.03 and 45.1 months in LUSC, respectively; Fig. 1g and h), although these differences were not significant.

### **EZH2** knockdown sensitizes EGFR-wild type (WT) LUAD cells to gefitinib and suppress cell viability and proliferation in vitro

**Fig. 2:**
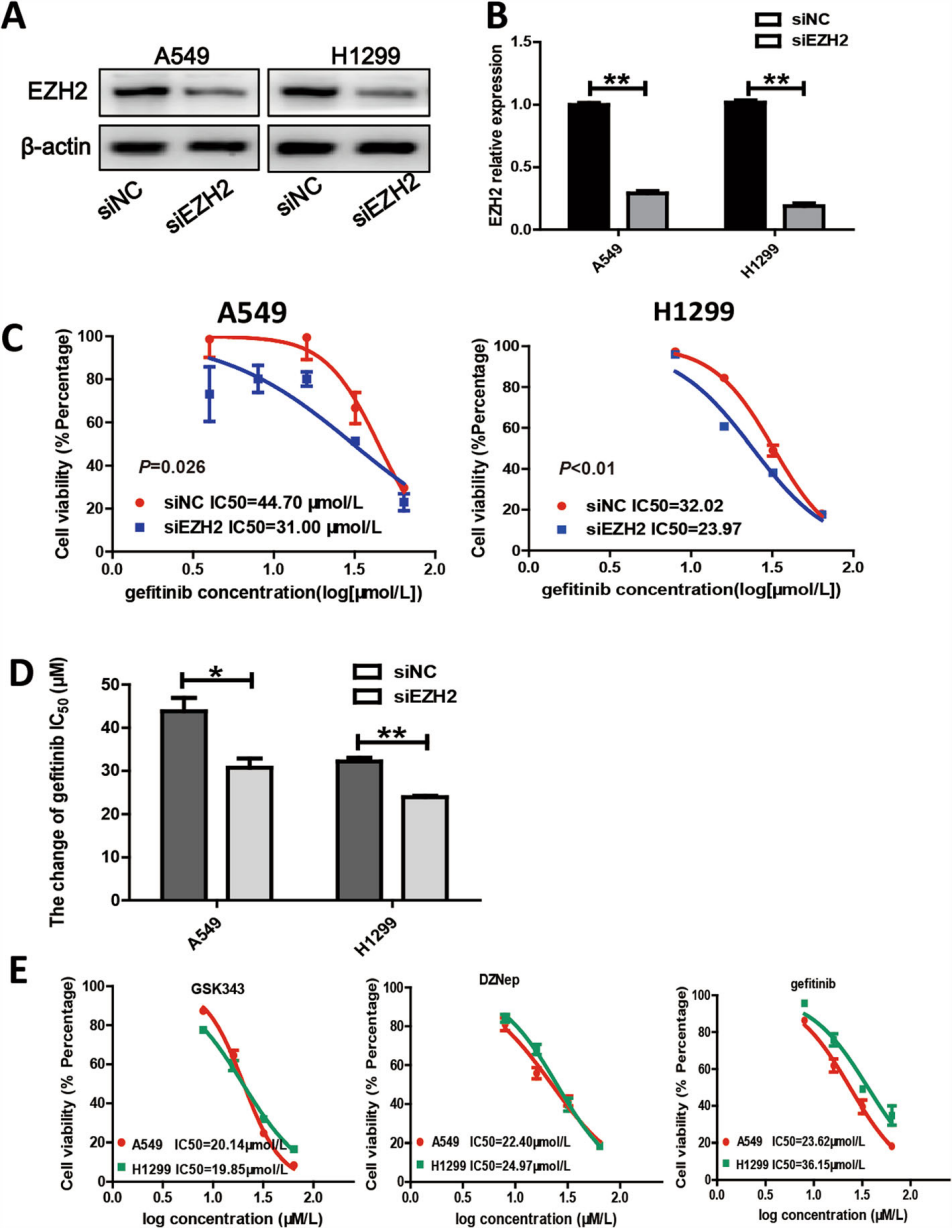
Knockdown of EZH2 increased the sensitivity to gefitinib in EGFR wild-type A549 and H1299 cells. **a** EZH2 protein levels were assessed by western blotting. **b** Quantitative PCR analysis of relative EZH2 mRNA expression level normalized to GAPDH expression level. **c-d** Cell proliferation of A549 and H1299 cells transfected with siNC or siEZH2 was measured using the CCK-8 assay. Data are representative of three independent experiments.**e** The IC50 of GSK343,DZNep and gefitinib were analysis by CCK-8 assay. All results are presented as mean ± standard deviation (SD) of triplicate experiments. * *P* < 0.05, ** *P* < 0.01 vs. the gefitinib alone-treated group

To test whether EZH2 affects the sensitivity of LUAD cells to EGFR-TKIs, we knocked down EZH2 expression in lung cancer cells using siRNA and subjected the cells to the CCK8 assay after exposure to different concentrations of gefitinib for 24–48 h. As shown in Fig. 2a and b, EZH2 expressions in A549 and H1299 cells were significantly knocked down by EZH2 siRNA. Using the CCK8 assay, the viability of these cells was compared with that of cells treated with scrambled control siRNA and different gefitinib concentrations. As shown in Fig. 2c and d, knockdown of EZH2 increased the cell sensitivity to gefitinib in A549 and H1299 cells, while siEZH2 was no obvious effect on the sensitivity to gefitinib in HCC827 cells (Fig. S1a). These results indicate that EZH2 knockdown sensitizes EGFR-WT LUAD cells to gefitinib.

**Fig. 3:**
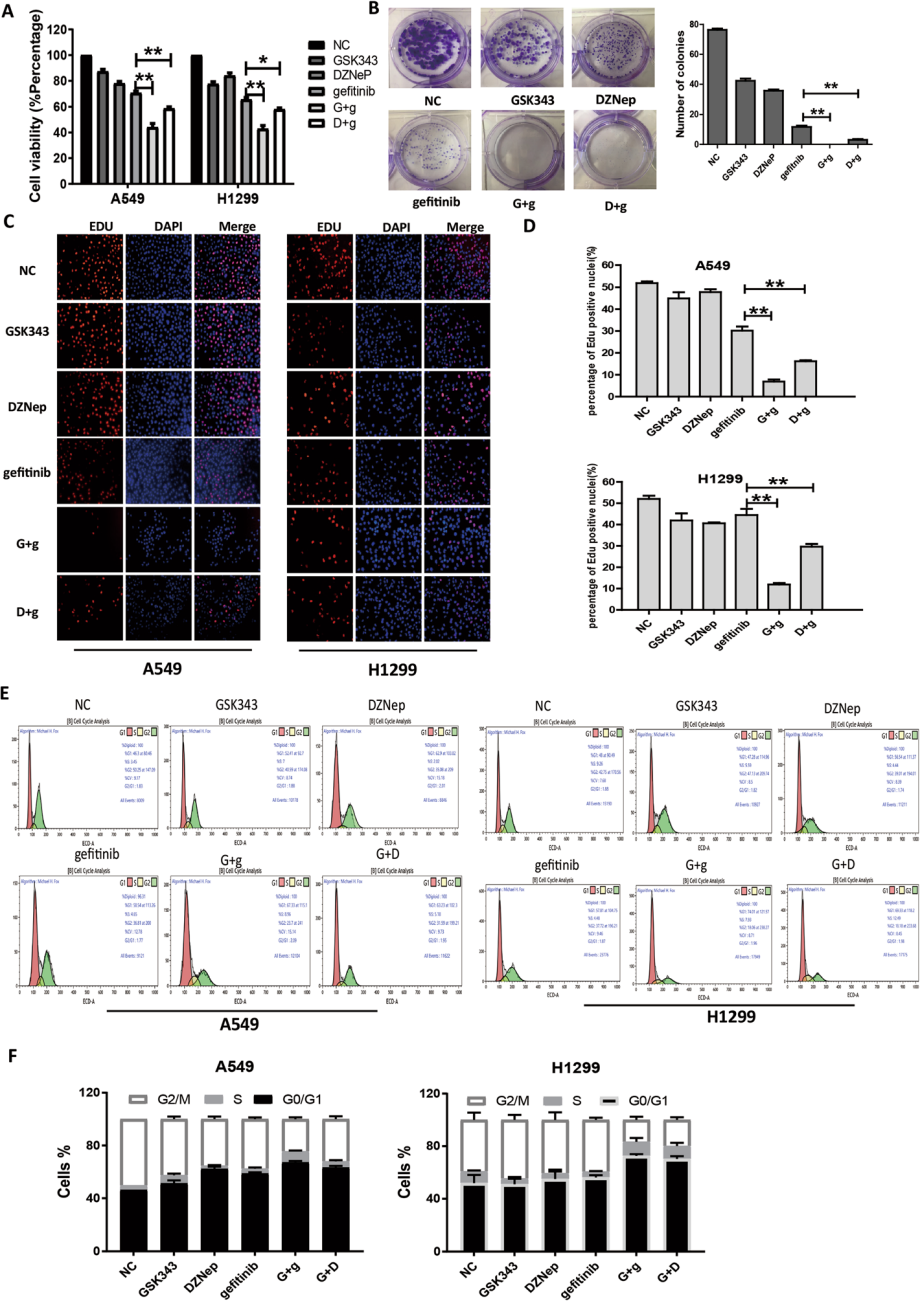
Co-administration of EZH2 inhibitors with gefitinib significantly suppresses lung cancer cell growth in vitro. Cells were subjected to control treatment (NC), GSK343 (11 μmol/L), DZNep (10 μmol/L), gefitinib (12 μmol/L), G + g (11 μmol/L GSK343 + 12 μmol/L gefitinib), or D + g (10 μmol/L DZNep + 12 μmol/L gefitinib) for 48 h. **a** Cell viabilities of A549 and H1299 cells were tested by CCK-8 assay. **b** The effects of different drug treatments on cell proliferation of A549 cells was measured by colony formation assay. **c-d** The effects of GSK343, DZNep, and gefitinib on cell proliferation of A549 and H1299 cells were evaluated by EDU assay.** *P* < 0.01 vs. the gefitinib alone-treated group. **e-f** Cell cycle of A549 and H1299 cells were analyzed by flow cytometry analysis. Data are presented as mean ± standard deviation

Next, DZNep and GSK343 were applied to A549, H1299 and HCC827 cells (Fig. 2e). To further determine whether these drugs enhanced the antitumor effects of gefitinib in EGFR-WT NSCLC cells, we studied the effects of EZH2 and EGFR co-inhibition on the viability of A549 and H1299 cells using the CCK8 assay. We divided the cells into NC (10% serum medium alone), GSK343 (11 μmol/L GSK343), DZNep (10 μmol/L DZNep), gefitinib (12 μmol/L gefitinib), G + g (11 μmol/L GSK343 + 12 μmol/L gefitinib), and D + g (10 μmol/L DZNep + 12 μmol/L gefitinib). CCK-8 assay revealed IC_50_ values of 20.14, 19.85, and 23.62 μmol/L for GSK343, DZNep, and gefitinib, respectively, in A549 cells, and corresponding values of 22.40, 24.97, and 36.15 μmol/L, respectively, in H1299 cells. The IC50 values of GSK343, DZNep, and gefitinib was 42.38, 169.8, and 2.906 μmol/L in HCC827 cells, respectively (Fig. S1b). The CCK-8 assay showed that compared with NC, A549 cell viability rate was 87.39 ± 2.68, 78.04 ± 2.69, 70.65 ± 2.35, 44.11 ± 5.09, and 58.75 ± 2.05 for GSK343, DZNep, gefitinib, G + g, and D + g, respectively; in H1299 cells, the corresponding values were 77.66 ± 2.79, 84.14 ± 3.43, 65.75 ± 2.5, 42.99 ± 4.86, and 58.14 ± 1.81, respectively. The results indicate that the co-administration of GSK343 with gefitinib more effectively reduced cell viability than administration of gefitinib alone (*P* < 0.01 for both cell lines), whereas the co-administration of DZNep with gefitinib inhibited cell viability more significantly (A549, *P* < 0.01; H1299, *P* < 0.05; Fig. 3a).

The colony formation assay was performed to determine the effects of drug co-inhibition on cell proliferation. The treatment group was divided into six groups as described above. Cells were subjected to different treatments according to the previously mentioned drug concentration for 48 h. As shown in Fig. 3b, 42.33 ± 2.52, 35.67 ± 1.53, 11.67 ± 1.53, 0 ± 0, and 3 ± 1 colonies were formed in GSK343, DZNep, gefitinib, G + g, and D + g and these values were significantly lower than those in NC (76.33 ± 1.53; *P* < 0.01).

The EdU assay was also performed to examine the effect of drugs treatment on cell proliferation (Fig. 3c and d). In A549 cells, the mean number of EdU-positive cells in NC, GSK343, gefitinib, DZNep, G + g, and D + g was 51.92 ± 1.64, 44.9 ± 6.97, 47.83 ± 3.2, 30.15 ± 4.67, 6.93 ± 2.36, and 16.16 ± 1.09, respectively. The combined effects of GSK343 + gefitinib or D + g on cell viability were greater than those of gefitinib alone (*P* < 0.01 for both). In H1299 cells, the mean number of EdU-positive cells in NC, GSK343, gefitinib, DZNep, G + g, and D + g was 52.07 ± 2.99, 41.93 ± 6.67, 40.63 ± 0.73, 44.47 ± 5.82, 11.95 ± 1.51, and 29.66 ± 3.13, respectively. These results showed that co-administration of EZH2 inhibitors with gefitinib significantly inhibited cell proliferation of A549 and H1299 cells,also HCC827 cells (Fig. S1c).

The effect of drug co-inhibition on cell cycle was evaluated by flow cytometry analysis. Results revealed that co-administration of EZH2 inhibitors with gefitinib could induced GO/G1 cell cycle arrest of A549 and H1299 cells (Fig. 3e and f).

### Co-administration of EZH2 inhibitors with gefitinib enhances the apoptosis of EGFR-WT A549 and H1299 cells

**Fig. 4:**
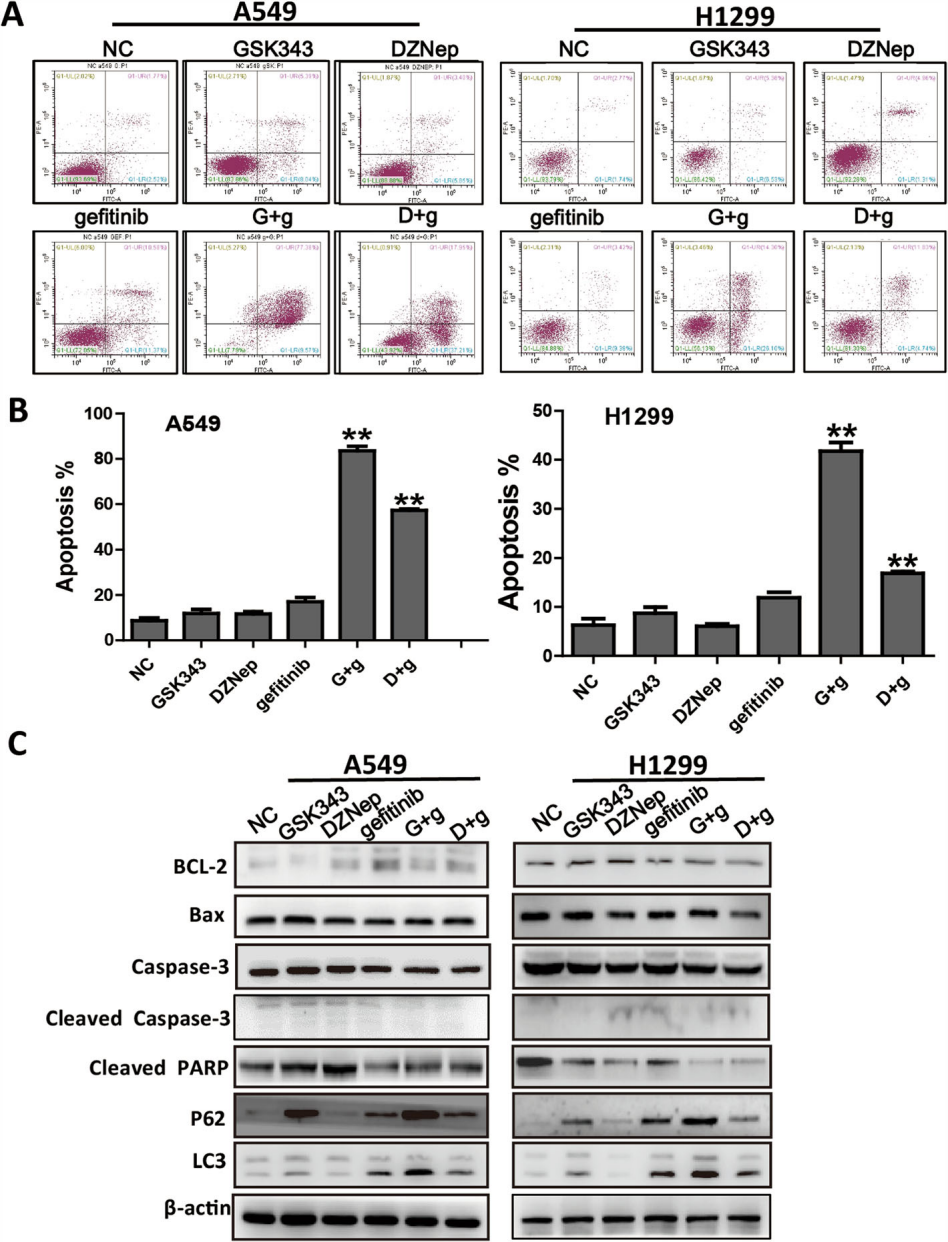
EZH2 inhibitors enhance gefitinib-induced apoptosis of primary gefitinib-resistant A549 and H1299 cell lines. **a**-**b** Apoptosis was analyzed by flow cytometry analysis after 48 h exposure to GSK343, DZNep, gefitinib, GSK343 + gefitinib, and D + g. Data are presented as mean ± standard deviation (*n* = 3 independent experiments).* *P* < 0.05, ** *P* < 0.01, vs. the gefitinib alone-treated group. **c** The effects of GSK343, DZNep, and gefitinib on Bcl-2, Bax, caspase-3, cleaved caspase-3 and PARP,p62 and LC3 protein levels in A549 and H1299 cells were evaluated by western blotting

Since the results showed that the co-administration of an EZH2 inhibitor with gefitinib sensitized the EGFR-WT NSCLC cells to gefitinib, we further investigated the influence of this treatment on cell apoptosis by flow cytometry analysis. As illustrated in Fig. 4a and b, the apoptosis rates of A549 cells in NC, GSK343, DZNep, gefitinib, G + g, and D + g groups were 8.59 ± 2.25, 11.9 ± 3.15, 11.63 ± 1.7, 17.03 ± 3.34, 83.51 ± 3.71, and 57.24 ± 0.98, respectively. And those apoptosis rates of H1299 cells were 6.32 ± 2.32, 8.71 ± 2.17, 6.08 ± 0.83, 11.91 ± 1.93, 41.77 ± 3.1, and 16.87 ± 0.6, respectively. These results showed that co-administration of EZH2 inhibitors with gefitinib significantly induced cell apoptosis of A549 and H1299 cells (Fig. 4a and b), but not HCC827 cell (Fig. S2a). However, neither GSK343 nor DZNep alone had a significant effect on apoptosis rate.

To study the effects of co-administration of EZH2 inhibitors on the apoptosis-related proteins, the levels of Bcl-2, Bax, caspase-3 and cleaved-PARP were detected by western blotting. As showed in Fig.4c, the expression of cleaved-PARP was decreased significantly in the co-administration of EZH2 with gefitinib group compared with the gefitinib alone-treated group in H1299 cells, while the levels of other apoptosis-related proteins were not change by co-administration of EZH2 inhibitors treatment in A549 and H1299 cells. We also investigate whether the co-administration of EZH2 inhibitors affect the autophagic activities of NSCLC cells. Interestingly, the lipidated LC3-II was significant increased in A549 and H1299 cells, while p62 was significant decreased in A549 cells treated with the co-administration of GSK343 with gefitinib compared with the gefitinib alone treated group (Fig. 4c). However, the levels of Bcl-2, caspase-3 and cleaved-PARP were no significant change in the co-administration of EZH2 inhibitors with gefitinib treatment group in HCC827 cells (Fig. S2b).

### Co-administration of EZH2 inhibitors with gefitinib inhibits EGFR-WT lung cancer cell migration in vitro

**Fig. 5:**
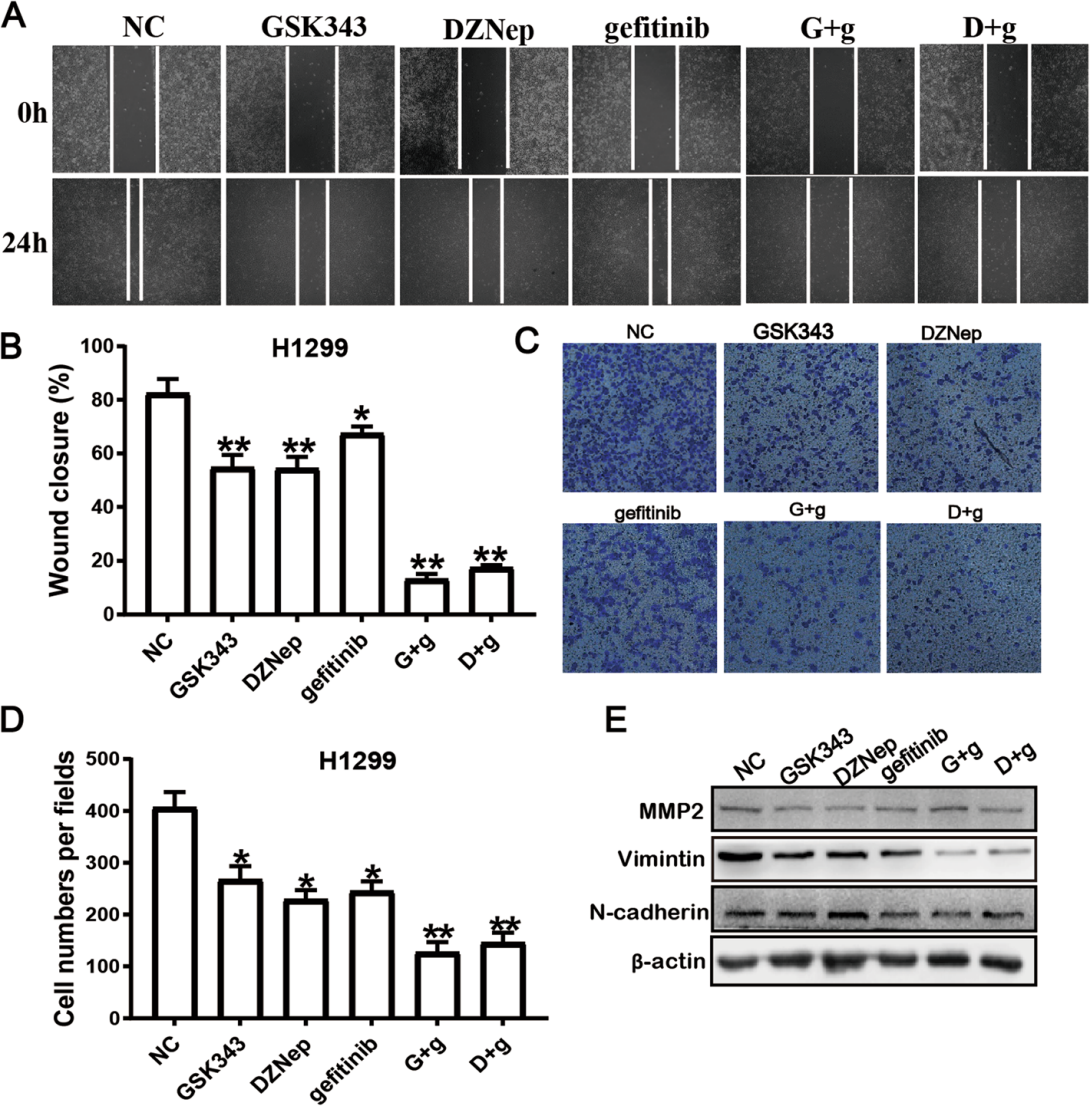
Combined treatment with EZH2 inhibitors and gefitinb inhibits EGFR wild-type NSCLC cell migration in vitro. **a-b** The effects of different drug treatments on cell migration of H1299 cells was detected by wound healing assay .Representative images are displayed at 4× magnification. Scale bar = 800 μm. Data are presented as mean ± standard deviation (SD). * *P* < 0.05, ** *P* < 0.01. **c-d** Transwell assay were performed to evaluate the migration ability of H1299 cells treated with different drug. Data are presented as mean ± SD. Scale bar = 200 μm, and images were are displayed at 20× magnification, * *P* < 0.05, ** *P* < 0.01. **e** The expression of MMP2, vimintin and N-cadherin in H1299 cells after treatment was detected by western blotting

We performed wound healing and transwell assays to explore the effects of co-administration of EZH2 inhibitor with gefitinib treatment on the migratory abilities of EGFR-WT NSCLC cells. Wound healing assay revealed that the migratory abilities of H1299 cells in GSK343, DZNep, and gefitinib significantly decreased compared with those of cells in NC (54.77 ± 4.71, 54.33 ± 4.35, and 67.47 ± 2.54 vs. 82.47 ± 5.3, *P* = < 0.01, < 0.01, and 0.01, respectively; Fig. 5a and b). The co-administration of GSK343 or DZNep with gefitinib had significantly stronger inhibitory effects on cell migratory ability than the administration of gefitinib alone (*P* < 0.01 for both). The rates of migrated cells in GSK343, DZNep, gefitinib, D + g, and G + g were 255 ± 12.99, 229 ± 18.19, 265.33 ± 21.03, 145.67 ± 19.5, and 126.67 ± 20.21, respectively. All combination groups had significantly greater decreases in cell migratory ability than NC (*P* < 0.01 for both; Fig. 5c and d). Taken together, our data demonstrate that EZH2 inhibitors (GSK343 and DZNep), when administered in combination with EGFR inhibitors, can inhibit the migration of EGFR-WT NSCLC cells.

We also investigate whether the co-administration of EZH2 inhibitors affect cell migration and invasion-related proteins. As showed in Fig. 5e, the expression of vimentin was decreased significantly in the co-administration of EZH2 with gefitinib group compared with the gefitinib alone-treated group in H1299 cells, while the levels of MMP2 and N-cadherin were not change by the co-administration of EZH2 with gefitinib in H1299 cells.

### Co-administration of drugs suppressed EZH2 and EGFR signaling pathways

**Fig. 6:**
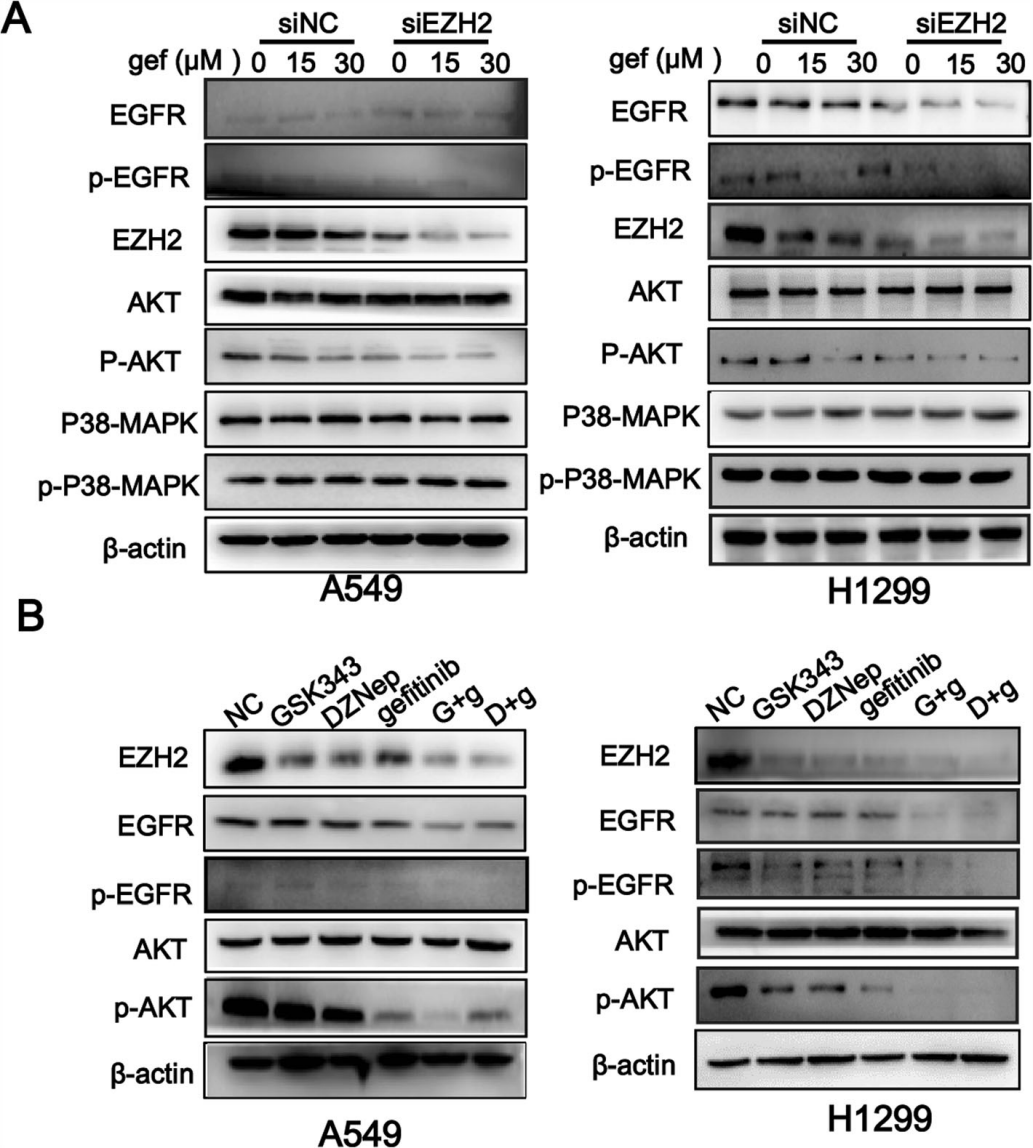
Co-administration of enhancer of zeste homolog 2 (EZH2) inhibitor with gefitinib suppresses EZH2 and the EGFR signaling pathways. Cells were treated with GSK343 (11 μmol/L), DZNep (10 μmol/L), gefitinib (12 μmol/L), G + g (11 μmol/L GSK343 + 12 μmol/L gefitinib), or D + g (10 μmol/L DZNep + 12 μmol/L gefitinib). **a** The effects of EZH2 silencing on the levels of the EZH2 and the key molecules of EGFR/AKT pathway (EGFR, AKT, and MAPK) in A549 and H1299 cells were evaluated by western blotting. **b** The effects of GSK343, DZNep, and gefitinib on the the EZH2, EGFR and AKT in A549 and H1299 cells were evaluated by western blotting

To explore the possible mechanisms underlying the above mentioned findings, we next evaluated the changes in EGFR signaling pathway-related proteins. As shown in Fig. 6a, A549 and H1299 cells were treated with different concentrations of gefitinib after EZH2 knockdown (siEZH2) and were subjected to western blotting. The results indicate that in both cell lines, co-administration of drugs slightly suppressed EGFR expression and strongly reduced EZH2 protein expression and EGFR phosphorylation levels. The phosphorylation of AKT, a downstream molecule of EGFR, was also inhibited by the co-administration of drugs. These results prove that gefitinib administration caused a concentrations-dependent decrease in the levels of p-EGFR, EZH2, and p-AKT, while the levels of AKT and EGFR were not significantly changed in A549 and H1299 cells. Meanwhile, more remarkable inhibition by gefitinib was observed in siEZH2 transfected A549 and H1299 cells. The levels of P38-MAPK and p-P38-MAPK, an important signaling pathway downstream of EGFR, were no significantly change in siNC and siEZH2 treated A549 and H1299 cells (Fig. 6a). However, markedly decreased EZH2 level, EGFR and AKT total and phosphorylation levels were observed in siNC and siEZH2 transfected HCC827 cells (Fig. S3a).

To explore how EZH2 silent affects cell’ sensitivity to gefitinib, GSK343 and DZNep were applied. As shown in Fig. 6b, markedly decreased EZH2 and EGFR total level, EGFR and AKT phosphorylation levels were observed in GSK343 and DZNep plus gefitinib group when compared with the gefitinib alone-treated group in A549 and H1299 cells. While in HCC827 cells, EGFR and EZH2 levels were observed to be decrease only in DZNep plus gefitinib group (Fig. S3b).

### Co-administration of GSK343 or DZNep with gefitinib suppresses the growth of EGFR-WT NSCLC in vivo

**Fig. 7:**
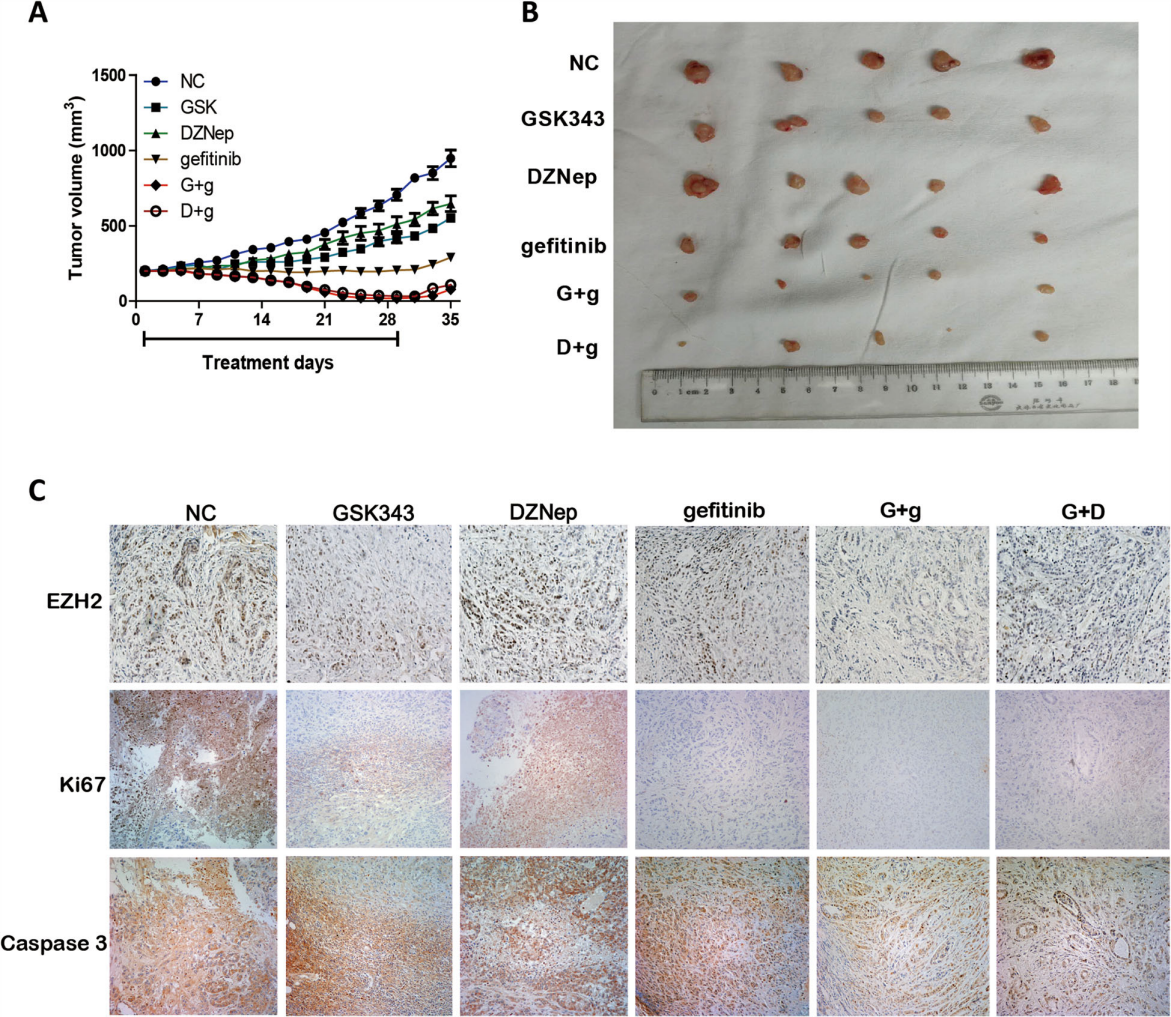
Co-administration of EZH2 inhibitors with gefitinib suppressed growth of H1299 cells in vivo. **a-b** GSK343, DZNep, gefitinib, GSK343 plus gefitinib and DZNep plus gefitinib and PBS were respectively injected subcutaneously into nude mice (*n* = 10). Tumor volumes in mice were measured every 5 days. Results are presented as mean ± standard deviation. * *P* < 0.05, ** *P* < 0.01. **c** Immunohistochemistry was performed to detect EZH2, ki67 and caspase-3 in tumor sections of nude mice

To further examine whether the co-administration of EZH2 inhibitors with gefitinib would inhibit tumor growth in vivo, we established a BALB/c mouse lung neoplasm xenograft model using A549 cells. As shown in Fig. 7a and b, GSK343, DZNep, and gefitinib mono-therapies all had inhibitory effects on tumor growth (*P* < 0.01 for all). However, the co-administration of GSK343 (4 mg/kg) or DZNep (2 mg/kg) with gefitinib (100 mg/kg) inhibited tumor growth significantly (*P* < 0.01, Fig. 7a). After 28 days of treatment, the combination groups had a tumor volume of < 280 mm^3^, and 10% (1/10) of the mice in G + g had been completely cured. No severe adverse effects were observed in any of the combination groups. To further investigate the mechanism underlying tumor suppression, we analyzed xenograft tumor sections using Immunohistochemistry to verify EZH2 protein expression level. As we expected, EZH2 levels were strongly reduced in G + g and D + g compared with in gefitinib (Fig. 7c). Ki67 and caspase-3 staining of the tumor sections showed decreases in cell proliferation as the percentage of Ki-67 and caspase-3 positive cells in the co-administration of GSK343 with gefitinib group. In conclusion, the above mentioned results indicate that the co-administration of EZH2 inhibitors with gefitinib inhibits lung cancer cell growth in vivo by inhibiting EZH2.

## Discussion

Lung cancer is among the most common and deadly cancers worldwide. NSCLC is the most common histological type of lung cancer, with LUSC and LUAD being the most common subtypes [20]. Although the techniques used to diagnose lung cancer and surgical, radiotherapeutic, and chemotherapeutic treatment methods have significantly improved, the 5-year OS rates of patients with lung cancer remain as low as approximately 15% [3, 15, 21, 22]. Tumor recurrence and metastasis present marked challenges to clinicians and severely affect the prognosis of these patients. Approximately 70% of patients with lung cancer experience different degrees of focal metastasis and local tumor recurrence after tumor resection and adjuvant treatment. The most important cause of recurrence and metastasis involves the development of different degrees of drug resistance such that the original drug treatment can no longer inhibit and kill the tumor cells, leading to further disease progression. Therefore, researchers must explore new therapeutic methods as well as develop new anticancer drugs. Molecular targeted drugs have revolutionized lung cancer treatment. EGFR-TKIs are the most representative class of targeted therapies. Compared with traditional chemotherapy, EGFR-TKIs effectively treat advanced NSCLC with EGFR mutations and significantly prolong the OS of those patients [23–25]. However, the application of EGFR-TKIs also has some certain limitations. First, there are restrictions in achieving the treatment objective because patients must have specific EGFR gene mutations to benefit from EGFR-TKI therapy. Second, during the 9- to 14-month period after a good initial response to an EGFR-TKI, most patients eventually develop drug resistance [26]. Recent studies have demonstrated that EGFR-TKIs also have a certain inhibitory effect on EGFR-WT cancer cells. Therefore, EGFR-TKIs are considered second-line treatment options for patients with EGFR-WT cancers [27–30]. Nevertheless, current mainstream research is focused on the suitability of EGFR-TKIs for EGFR-WT cancers and whether these drugs can be combined with other drugs to enhance their efficacy. In this study, we determined that gefitinib, a representative first-generation EGFR-TKI, can still exert anticancer effects in EGFR-WT NSCLC cells. These drugs not only inhibit cell viability but also inhibit cell migration and induce apoptosis in EGFR-WT A549 and H1299 cells; moreover, these effects can be enhanced by the co-administration of EZH2 inhibitors with gefitinib.

Developments in molecular biology and epigenetics have enabled researchers to determine that some special epigenetic trait changes (e.g., gene methylation and acetylation) will be present in tumors even if specific genetic locus changes (such as *KRAS* mutations) are excluded [31–33]. Epigenetic regulation plays an important role in cell growth, proliferation, differentiation, and apoptosis as well as an irreplaceable role in tumorigenesis and tumor development [34–36]. EZH2 was identified as an important epigenetic regulatory gene that regulates histone methyltransferase activity and methylation modification. EZH2 was originally identified in *Zeste*. Continuous exploration later revealed the presence of EZH2 in the human body, and abnormal EZH2 expression has been frequently detected in a range of solid tumors including prostate cancer, breast cancer, kidney cancer, lung cancer, and lymphoma [37, 38]. EZH2 is a polycomb protein homologous to the *Drosophila* enhancer of *zeste* and catalyzes the addition of methyl groups to H3K27. It plays important roles in tumorigenesis and cancer progression via epigenetic gene silencing and chromatin remodeling. EZH2 overexpression has been reported in various human malignancies including NSCLC and may be associated with worse outcomes [11, 39–42]. Numerous studies have analyzed the correlation between EZH2 expression and lung cancer prognosis. The general consensus is that EZH2 expression level is negatively correlates with the prognosis of patients with NSCLC [17, 43–46]. In this study, we determined the differences in EZH2 expression levels between paired tumor and paracancerous lung tissues using the TCGA database. When compared with paracancerous lung tissues, LUAD and LUSC expressed higher EZH2 expression levels. The results of TCGA database analysis also indicated that a higher EZH2 expression level was related to poor prognosis only in LUAD, which is inconsistent with the findings of previous studies [45, 47]. In addition, our clinical data analysis revealed a trend toward shorter OS in patients with higher EZH2 expression levels.

Given the important roles of EZH2 in tumorigenesis and development, researchers have developed various EZH2 inhibitors and have evaluated these through in vitro experiments and clinical studies. EZH2 inhibitors can enhance the sensitivity of tumor cells to antitumor drugs and thereby enhance the efficacy of the drugs. EZH2 has been reported to play an important role in the acquired resistance of tumor cells to chemotherapeutic drugs in small-cell lung cancer. For example, Gardner et al. reported that chemotherapeutic drugs induced the accumulation of H3K27me3 on SLFN11 via the methylation of EZH2, which led to partial chromatin condensation and inhibited EZH2 expression [48]. In NSCLC, EZH2 inhibitors could effectively enhance the sensitivity to etoposide in patients with BRG1- and EGFR-mutant lung cancers [44]. The inhibition of EZH2 expression could effectively reverse resistance to platinum-based chemotherapy in NSCLC [49]. The combination of the EZH2 inhibitor DZNep and histone deacetylase inhibitor Novelitar could significantly suppress NSCLC cell proliferation and induced apoptosis [18]. EZH2 inhibitors also enhanced sensitivity to soracinib in hepatoma carcinoma cells [50]. In this study, we found that EZH2 silencing enhanced gefitinib sensitivity in gefitinib-resistant cells. To further investigate the effects of the combination of an EZH2 inhibitor and gefitinib on primary gefitinib-resistant cells, we treated the EGFR-WT NSCLC cell lines A549 and H1299 with the EZH2 inhibitors DZNep and GSK343. The co-administration of either inhibitor with gefitinib more strongly inhibited cell proliferation and migration than any single drug alone. In addition, the co-treatment significantly inhibited the phosphorylation of AKT, which is activated downstream of EGFR. We also found that the co-administration of EZH2 inhibitors with gefitinib exerted good tumor-suppressing effects against primary gefitinib-resistant cells in vivo.

Lung cancer remains a life-threatening malignancy in humans. EGFR-targeted drugs have provided new treatment options for these tumors. EZH2 inhibitors have also provided new treatment concepts. In this study, we found that a therapeutic combination of an EZH2 inhibitor and gefitinib could significantly inhibit tumor growth and metastasis in primary gefitinib-resistant cells both in vivo and in vitro. Our findings indicate a new direction for the future clinical treatment of lung cancer.

## Conclusions

In summary, our study reveal that the co-administration of EZH2 inhibitors with EGFR-TKIs may be feasible for the treatment of EGFR wild-type (WT) NSCLC in patients who refuse traditional chemotherapy.

## Supplementary Information

**Additional file 1: Fig. S1**. The effort of enhancer of zeste homolog 2 (EZH2) inhibitors with gefitinib on cell growth of HCC827 cells. a Cell proliferation of cells transfected with siEZH2 or siNC was measured using the CCK-8 assay.**b** The IC50 of gefitinib, GSK343 and DZNep were analysis by CCK-8 assay.**c** The effects of GSK343, DZNep, and gefitinib on cell proliferation of A549 and H1299 cells were evaluated by EDU assay. Data are presented as mean ± standard deviation (*n* = 3 independent experiments). * *P* < 0.05 vs. the gefitinib alone-treated group.

**Additional file 2: Fig. S2**. The effort of enhancer of zeste homolog 2 (EZH2) inhibitors with gefitinib on cell apoptosis of HCC827 cells. **a** Apoptosis was analyzed by flow cytometry analysis after 48 h of exposure to GSK343, DZNep, gefitinib, GSK343 + gefitinib, and D + g in HCC827 cells. Data are presented as mean ± standard deviation (*n* = 3 independent experiments). **b** The effects of GSK343, DZNep, and gefitinib on Bcl-2, and caspase-3 protein levels in HCC827 cells were evaluated by western blotting.

**Additional file 3: Fig. S3**. The effort of enhancer of zeste homolog 2 (EZH2) inhibitors with gefitinib on EZH2 and the EGFR signaling pathways in HCC827 cells.**a** The effects of EZH2 knockdown on the levels of EZH2 and the key molecules of EGFR/AKT pathway (EGFR, AKT, and MAPK) in HCC827 cells were evaluated by western blotting.**b** The effects of GSK343, DZNep, and gefitinib on the levels of EZH2,EGFR and AKT in HCC827 cells were evaluated by western blotting.

## Data Availability

All data generated or analyzed during this study are included in this published article.

## Abbreviations

NSCLC: Non-small-cell lung cancer
LUAD: Lung adenocarcinoma
LUSC: Lung squamous cell carcinoma
EGFR-TKI: Epidermal growth factor receptor (EGFR)-tyrosine kinase inhibitor
KRAS: Kirsten rat sarcoma viral proto-oncogene
EZH2: Enhancer of zeste homolog 2
PRC2: Polycomb suppression complex 2;TCGA: The Cancer Genome Atlas
wt: wild-type
OS: Overall survival
TPM: Transcripts per million

## References

[1] Bray F, Ferlay J, Soerjomataram I, Siegel RL, Torre LA, Jemal A (2018). Global cancer statistics 2018: GLOBOCAN estimates of incidence and mortality worldwide for 36 cancers in 185 countries. CA Cancer J Clin.

[2] Torre LA, Bray F, Siegel RL, Ferlay J, Lortet-Tieulent J, Jemal A (2015). Global cancer statistics, 2012. CA Cancer J Clin.

[3] Siegel RL, Miller KD, Jemal A (2018). Cancer statistics, 2018. CA Cancer J Clin.

[4] Verdecchia A, Francisci S, Brenner H, Gatta G, Micheli A, Mangone L, Kunkler I (2007). EUROCARE-4 working group. Recent cancer survival in Europe: a 2000-02 period analysis of EUROCARE-4 data. Lancet Oncol.

[5] Wang Z, Candelora C (2017). In vitro enzyme kinetics analysis of EGFR. Methods Mol Biol.

[6] Nan X, Xie C, Yu X, Liu J (2017). EGFR TKI as first-line treatment for patients with advanced EGFR mutation-positive non-small-cell lung cancer. Oncotarget..

[7] Morgillo F, Della Corte CM, Fasano M, Ciardiello F (2016). Mechanisms of resistance to EGFR-targeted drugs: lung cancer. ESMO Open.

[8] Zhou Q, Cheng Y, Yang JJ, Zhao MF, Zhang L, Zhang XC, Chen ZH, Yan HH, Song Y, Chen JH (2014). Pemetrexed versus gefitinib as a second-line treatment in advanced nonsquamous nonsmall-cell lung cancer patients harboring wild-type EGFR (CTONG0806): a multicenter randomized trial. Ann Oncol.

[9] Sequist LV, Yang JC, Yamamoto N, O'Byrne K, Hirsh V, Mok T, Geater SL, Orlov S, Tsai CM, Boyer M (2013). Phase III study of afatinib or cisplatin plus pemetrexed in patients with metastatic lung adenocarcinoma with EGFR mutations. J Clin Oncol.

[10] Margueron R, Reinberg D (2011). The Polycomb complex PRC2 and its mark in life. Nature..

[11] Behrens C, Solis LM, Lin H, Yuan P, Tang X, Kadara H, Riquelme E, Galindo H, Moran CA, Kalhor N (2013). EZH2 protein expression associates with the early pathogenesis, tumor progression, and prognosis of non-small cell lung carcinoma. Clin Cancer Res.

[12] Huqun I (2012). R, Zhang J, Miyazawa H, Goto Y, Shimizu Y, Hagiwara K, Koyama N. enhancer of zeste homolog 2 is a novel prognostic biomarker in nonsmall cell lung cancer. Cancer..

[13] Zhang Y, Lin C, Liao G, Liu S, Ding J, Tang F, Wang Z, Liang X, Li B, Wei Y (2015). MicroRNA-506 suppresses tumor proliferation and metastasis in colon cancer by directly targeting the oncogene EZH2. Oncotarget..

[14] Chase A, Cross NC (2011). Aberrations of EZH2 in cancer. Clin Cancer Res.

[15] Xu C, Hou Z, Zhan P, Zhao W, Chang C, Zou J, Hu H, Zhang Y, Yao X, Yu L (2013). EZH2 regulates cancer cell migration through repressing TIMP-3 in non-small cell lung cancer. Med Oncol.

[16] Kikuchi J, Kinoshita I, Shimizu Y, Kikuchi E, Konishi J, Oizumi S, Kaga K, Matsuno Y, Nishimura M, Dosaka-Akita H (2010). Distinctive expression of the polycomb group proteins Bmi1 polycomb ring finger oncogene and enhancer of zeste homolog 2 in nonsmall cell lung cancers and their clinical and clinicopathologic significance. Cancer..

[17] Takashina T, Kinoshita I, Kikuchi J, Shimizu Y, Sakakibara-Konishi J, Oizumi S, Nishimura M, Dosaka-Akita H (2016). Combined inhibition of EZH2 and histone deacetylases as a potential epigenetic therapy for non-small-cell lung cancer cells. Cancer Sci.

[18] Miranda TB, Cortez CC, Yoo CB, Liang G, Abe M, Kelly TK, Marquez VE, Jones PA (2009). DZNep is a global histone methylation inhibitor that reactivates developmental genes not silenced by DNA methylation. Mol Cancer Ther.

[19] Sun J, Nishiyama T, Shimizu K, Kadota K (2013). TCC: an R package for comparing tag count data with robust normalization strategies. BMC Bioinformatics.

[20] Herbst RS, Heymach JV, Lippman SM (2008). Lung cancer. N Engl J Med.

[21] Duma N, Santana-Davila R, Molina JR (2019). Non-small cell lung Cancer: epidemiology, screening, diagnosis, and treatment. Mayo Clin Proc.

[22] Liao Y, Fan X, Wang X (2019). Effects of different metastasis patterns, surgery and other factors on the prognosis of patients with stage IV non-small cell lung cancer: a surveillance, epidemiology, and end results (SEER) linked database analysis. Oncol Lett.

[23] Wang C, Wang T, Lv D, Li L, Yue J, Chen HZ, Xu L (2019). Acquired resistance to EGFR TKIs mediated by TGFbeta1/integrin beta3 signaling in EGFR-mutant lung Cancer. Mol Cancer Ther.

[24] Saida Y, Watanabe S, Abe T, Shoji S, Nozaki K, Ichikawa K, Kondo R, Koyama K, Miura S, Tanaka H (2019). Efficacy of EGFR-TKIs with or without upfront brain radiotherapy for EGFR-mutant NSCLC patients with central nervous system metastases. Thorac Cancer.

[25] Cheng H, Li XJ, Wang XJ, Chen ZW, Wang RQ, Zhong HC, Wu TC, Cao QD (2019). A meta-analysis of adjuvant EGFR-TKIs for patients with resected non-small cell lung cancer. Lung Cancer.

[26] Liu M, Xu S, Wang Y, Li Y, Li Y, Zhang H, Liu H, Chen J (2016). PD 0332991, a selective cyclin D kinase 4/6 inhibitor, sensitizes lung cancer cells to treatment with epidermal growth factor receptor tyrosine kinase inhibitors. Oncotarget..

[27] Cross DA, Ashton SE, Ghiorghiu S, Eberlein C, Nebhan CA, Spitzler PJ, Orme JP, Finlay MR, Ward RA, Mellor MJ (2014). AZD9291, an irreversible EGFR TKI, overcomes T790M-mediated resistance to EGFR inhibitors in lung cancer. Cancer Discov.

[28] Walter AO, Sjin RT, Haringsma HJ, Ohashi K, Sun J, Lee K, Dubrovskiy A, Labenski M, Zhu Z, Wang Z (2013). Discovery of a mutant-selective covalent inhibitor of EGFR that overcomes T790M-mediated resistance in NSCLC. Cancer Discov.

[29] Tjin Tham Sjin R, Lee K, Walter AO, Dubrovskiy A, Sheets M, Martin TS, Labenski MT, Zhu Z, Tester R, Karp R (2014). In vitro and in vivo characterization of irreversible mutant-selective EGFR inhibitors that are wild-type sparing. Mol Cancer Ther.

[30] Paz-Ares LG, de Marinis F, Dediu M, Thomas M, Pujol JL, Bidoli P, Molinier O, Sahoo TP, Laack E, Reck M (2013). PARAMOUNT: final overall survival results of the phase III study of maintenance pemetrexed versus placebo immediately after induction treatment with pemetrexed plus cisplatin for advanced nonsquamous non-small-cell lung cancer. J Clin Oncol.

[31] Decourcelle A, Leprince D, Dehennaut V (2019). Regulation of Polycomb Repression by O-GlcNAcylation: Linking Nutrition to Epigenetic Reprogramming in Embryonic Development and Cancer. Front Endocrinol (Lausanne).

[32] Jiao J, Sagnelli M, Shi B, Fang Y, Shen Z, Tang T, Dong B, Li D, Wang X (2019). Genetic and epigenetic characteristics in ovarian tissues from polycystic ovary syndrome patients with irregular menstruation resemble those of ovarian cancer. BMC Endocr Disord.

[33] Fritz AJ, Gillis NE, Gerrard DL, Rodriguez PD, Hong D, Rose JT, Ghule PN, Bolf EL, Gordon JA, Tye CE (2019). Higher order genomic organization and epigenetic control maintain cellular identity and prevent breast cancer. Genes Chromosom Cancer.

[34] Cai L, Bai H, Duan J, Wang Z, Gao S, Wang D, Wang S, Jiang J, Han J, Tian Y (2019). Epigenetic alterations are associated with tumor mutation burden in non-small cell lung cancer. J Immunother Cancer.

[35] Zhang L (2019). Linking metabolic and epigenetic regulation in the development of lung cancer driven by TGFbeta signaling. AIMS Genet.

[36] Naqvi SMH, Kim Y (2019). Epigenetic modification by galactic cosmic radiation as a risk factor for lung cancer: real world data issues. Transl Lung Cancer Res.

[37] Yu Y, Qi J, Xiong J, Jiang L, Cui D, He J, Chen P, Li L, Wu C, Ma T (2019). Epigenetic Co-Deregulation of EZH2/TET1 is a Senescence-Countering, Actionable Vulnerability in Triple-Negative Breast Cancer. Theranostics.

[38] Batool A, Jin C, Liu YX (2019). Role of EZH2 in cell lineage determination and relative signaling pathways. Front Biosci.

[39] McCabe MT, Mohammad HP, Barbash O, Kruger RG (2017). Targeting histone methylation in Cancer. Cancer J.

[40] Park KS, Kim HK, Lee JH, Choi YB, Park SY, Yang SH, Kim SY, Hong KM (2010). Transglutaminase 2 as a cisplatin resistance marker in non-small cell lung cancer. J Cancer Res Clin Oncol.

[41] Hussein YR, Sood AK, Bandyopadhyay S, Albashiti B, Semaan A, Nahleh Z, Roh J, Han HD, Lopez-Berestein G, Ali-Fehmi R (2012). Clinical and biological relevance of enhancer of zeste homolog 2 in triple-negative breast cancer. Hum Pathol.

[42] Xu C, Hao K, Hu H, Sheng Z, Yan J, Wang Q, Yu L (2014). Expression of the enhancer of zeste homolog 2 in biopsy specimen predicts chemoresistance and survival in advanced non-small cell lung cancer receiving first-line platinum-based chemotherapy. Lung Cancer.

[43] Ihira K, Dong P, Xiong Y, Watari H, Konno Y, Hanley SJ, Noguchi M, Hirata N, Suizu F, Yamada T (2017). EZH2 inhibition suppresses endometrial cancer progression via miR-361/twist axis. Oncotarget..

[44] Fillmore CM, Xu C, Desai PT, Berry JM, Rowbotham SP, Lin YJ, Zhang H, Marquez VE, Hammerman PS, Wong KK (2015). EZH2 inhibition sensitizes BRG1 and EGFR mutant lung tumours to TopoII inhibitors. Nature..

[45] Wang X, Zhao H, Lv L, Bao L, Wang X, Han S (2016). Prognostic significance of EZH2 expression in non-small cell lung Cancer: a meta-analysis. Sci Rep.

[46] Li Z, Xu L, Tang N, Xu Y, Ye X, Shen S, Niu X, Lu S, Chen Z (2014). The polycomb group protein EZH2 inhibits lung cancer cell growth by repressing the transcription factor Nrf2. FEBS Lett.

[47] Kim NY, Pyo JS (2017). Clinicopathological significance and prognostic role of EZH2 expression in non-small cell lung cancer. Pathol Res Pract.

[48] Gardner EE, Lok BH, Schneeberger VE, Desmeules P, Miles LA, Arnold PK, Ni A, Khodos I, de Stanchina E, Nguyen T (2017). Chemosensitive relapse in small cell lung Cancer proceeds through an EZH2-SLFN11 Axis. Cancer Cell.

[49] Liu H, Li W, Yu X, Gao F, Duan Z, Ma X, Tan S, Yuan Y, Liu L, Wang J (2016). EZH2-mediated Puma gene repression regulates non-small cell lung cancer cell proliferation and cisplatin-induced apoptosis. Oncotarget..

[50] Follis AV, Chipuk JE, Fisher JC, Yun MK, Grace CR, Nourse A, Baran K, Ou L, Min L, White SW (2013). PUMA binding induces partial unfolding within BCL-xL to disrupt p53 binding and promote apoptosis. Nat Chem Biol.

