# Supplementary figures and images for: EZH2 inhibitors reverse resistance to gefitinib in primary EGFR wild-type lung cancer cells
Source: BMC Cancer. 2020 Dec 4;20:1189. doi: 10.1186/s12885-020-07667-7 (PMC7716470; doi:10.1186/s12885-020-07667-7)

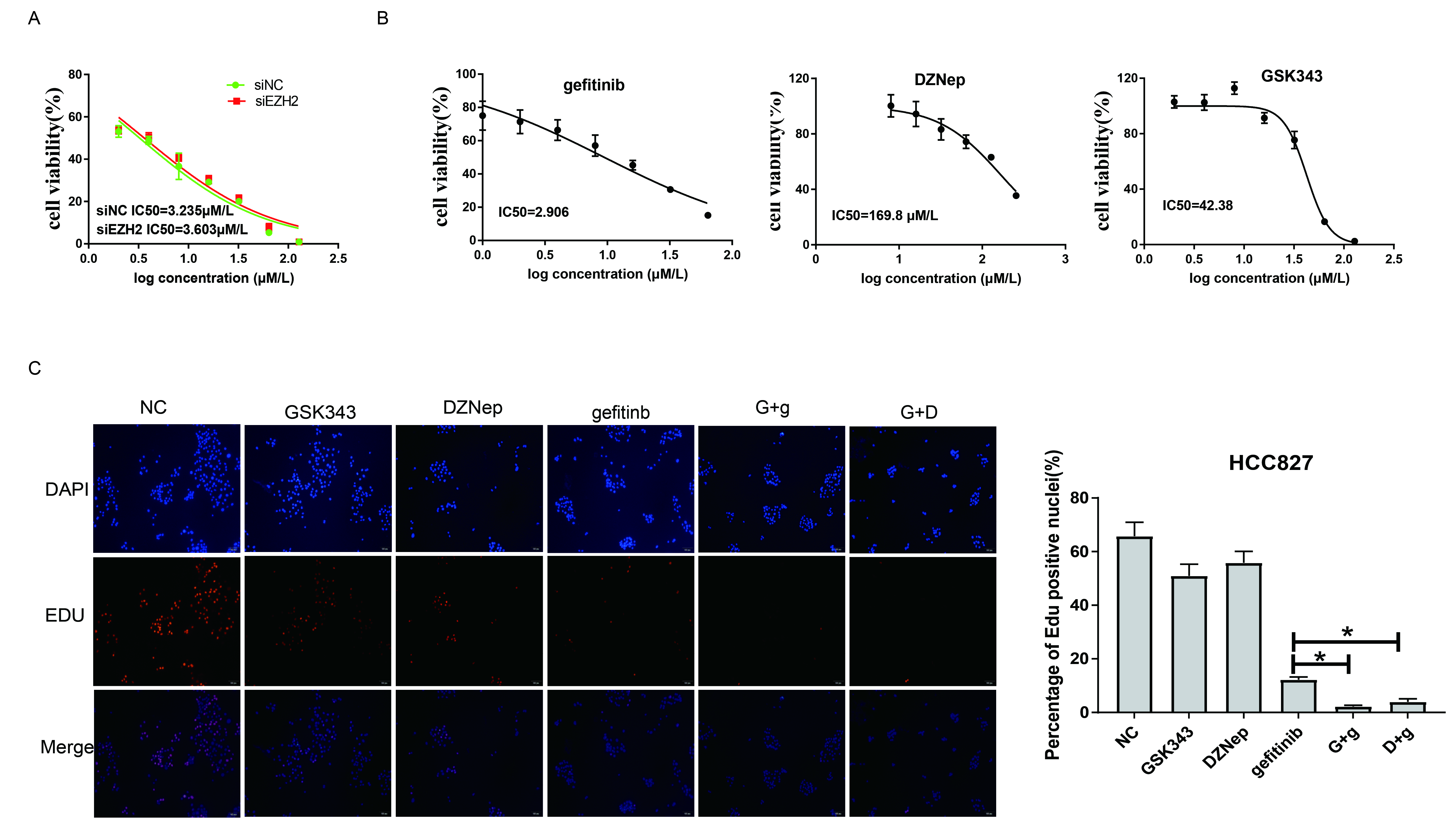

Supplement: Supplementary file 1 — Additional file 1: Fig. S1. The effort of enhancer of zeste homolog 2 (EZH2) inhibitors with gefitinib on cell growth of HCC827 cells. a Cell proliferation of cells transfected with siEZH2 or siNC was measured using the CCK-8 assay.b The IC50 of gefitinib, GSK343 and DZNep were analysis by CCK-8 assay.c The effects of GSK343, DZNep, and gefitinib on cell proliferation of A549 and H1299 cells were evaluated by EDU assay. Data are presented as mean ± standard deviation (n = 3 independent experiments). * P < 0.05 vs. the gefitinib alone-treated group. [file 12885_2020_7667_MOESM1_ESM.tif]

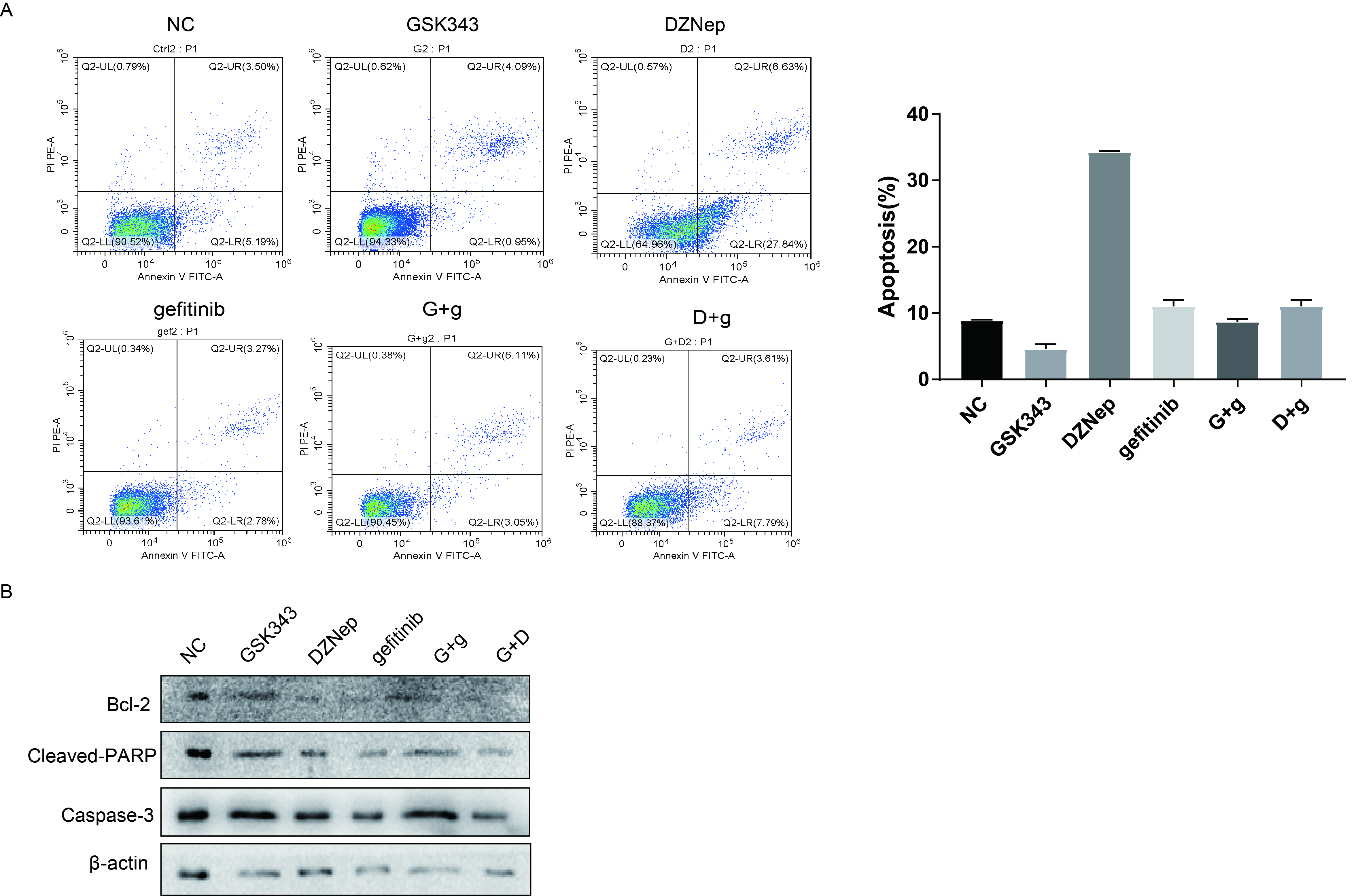

Supplement: Supplementary file 2 — Additional file 2: Fig. S2. The effort of enhancer of zeste homolog 2 (EZH2) inhibitors with gefitinib on cell apoptosis of HCC827 cells. a Apoptosis was analyzed by flow cytometry analysis after 48 h of exposure to GSK343, DZNep, gefitinib, GSK343 + gefitinib, and D + g in HCC827 cells. Data are presented as mean ± standard deviation (n = 3 independent experiments). b The effects of GSK343, DZNep, and gefitinib on Bcl-2, and caspase-3 protein levels in HCC827 cells were evaluated by western blotting. [file 12885_2020_7667_MOESM2_ESM.tif]

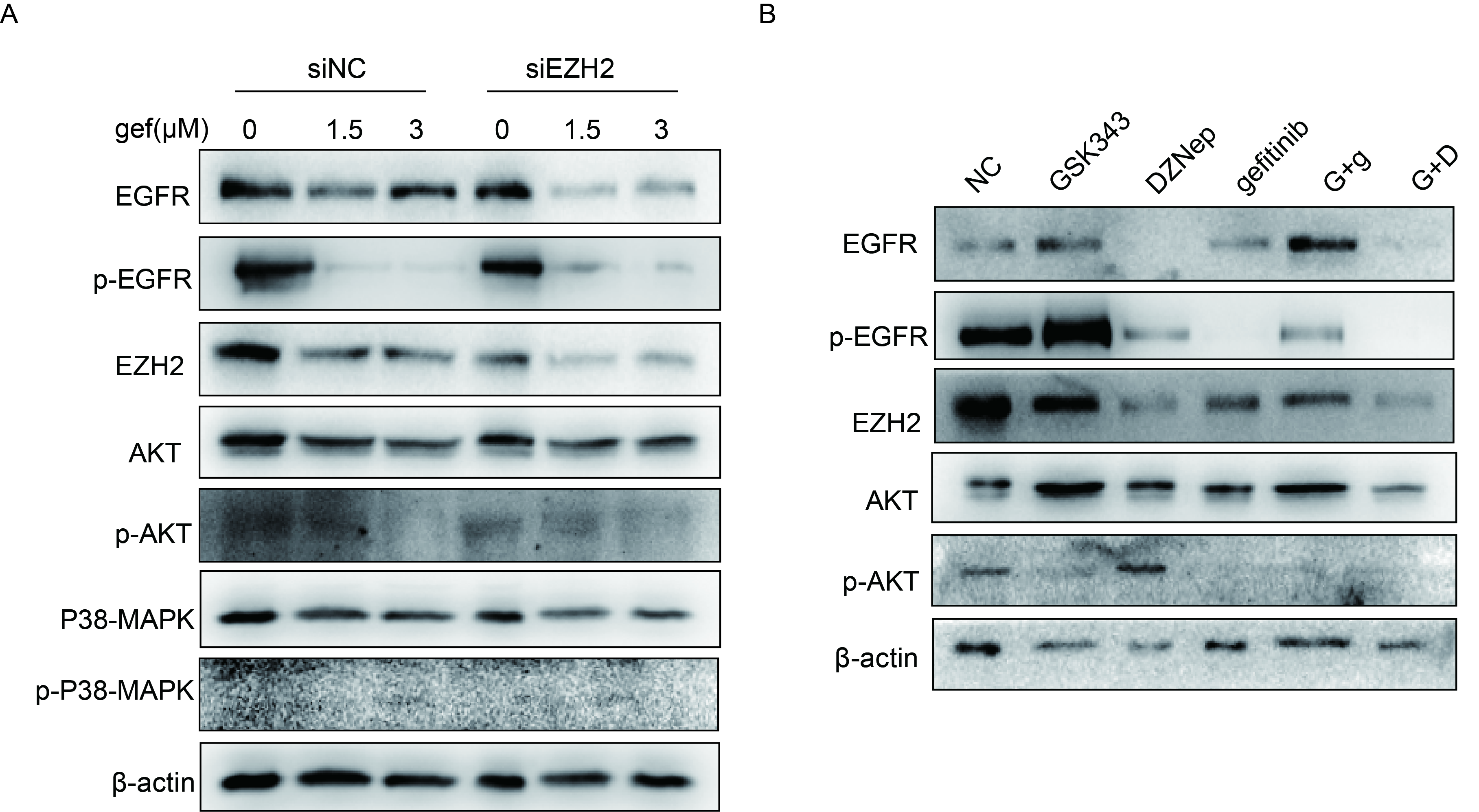

Supplement: Supplementary file 3 — Additional file 3: Fig. S3. The effort of enhancer of zeste homolog 2 (EZH2) inhibitors with gefitinib on EZH2 and the EGFR signaling pathways in HCC827 cells.a The effects of EZH2 knockdown on the levels of EZH2 and the key molecules of EGFR/AKT pathway (EGFR, AKT, and MAPK) in HCC827 cells were evaluated by western blotting.b The effects of GSK343, DZNep, and gefitinib on the levels of EZH2,EGFR and AKT in HCC827 cells were evaluated by western blotting. [file 12885_2020_7667_MOESM3_ESM.tif]
